# Decoding the Roles of Amyloid-β (1–42)’s Key Oligomerization Domains toward Designing Epitope-Specific Aggregation Inhibitors

**DOI:** 10.1021/jacsau.2c00668

**Published:** 2023-03-02

**Authors:** Dongjoon Im, Soohyeong Kim, Gyusub Yoon, Da Gyeong Hyun, Yu-Gon Eom, Ye Eun Lee, Chang Ho Sohn, Jeong-Mo Choi, Hugh I. Kim

**Affiliations:** † Department of Chemistry, Korea University, Seoul 02841, Republic of Korea; ‡ Center for Proteogenome Research, Korea University, Seoul 02841, Republic of Korea; § Single Cell Analysis Laboratory, Korea University, Seoul 02841, Republic of Korea; ∥ Department of Chemistry, Pusan National University, Busan 46241, Republic of Korea; ⊥ Center for Nanomedicine, Institute for Basic Science (IBS), Seoul 03722, Republic of Korea; # Graduate Program in Nanobiomedical Engineering, Advanced Science Institute, Yonsei University, Seoul 03722, Republic of Korea; ∇ Chemistry Institute for Functional Materials, Pusan National University, Busan 46241, Republic of Korea

**Keywords:** peptides and proteins, conformation, oligomers, aggregation, inhibitors

## Abstract

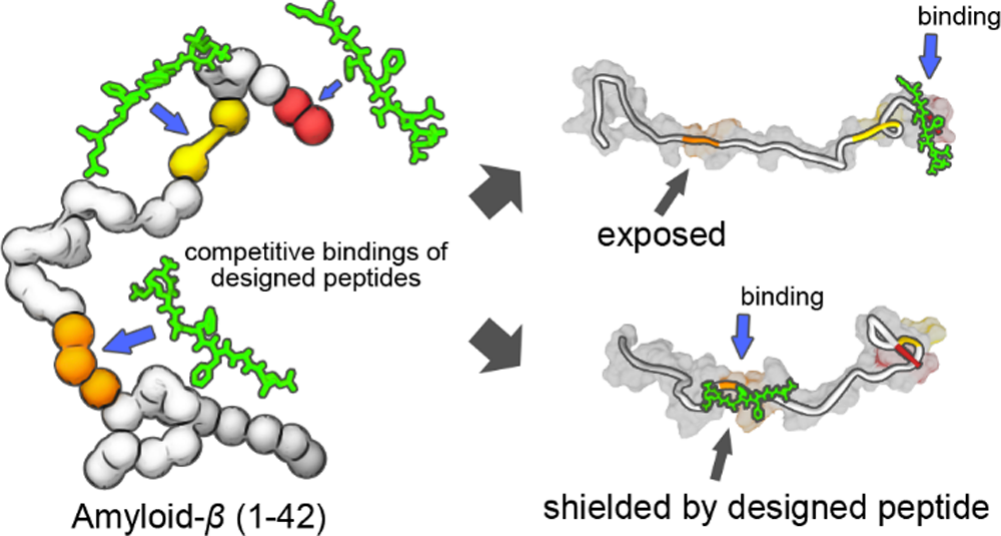

Fibrillar amyloid aggregates are the pathological hallmarks of multiple neurodegenerative diseases. The amyloid-β (1–42) protein, in particular, is a major component of senile plaques in the brains of patients with Alzheimer’s disease and a primary target for disease treatment. Determining the essential domains of amyloid-β (1–42) that facilitate its oligomerization is critical for the development of aggregation inhibitors as potential therapeutic agents. In this study, we identified three key hydrophobic sites (^17^LVF^19^, ^32^IGL^34^, and ^41^IA^42^) on amyloid-β (1–42) and investigated their involvement in the self-assembly process of the protein. Based on these findings, we designed candidate inhibitor peptides of amyloid-β (1–42) aggregation. Using the designed peptides, we characterized the roles of the three hydrophobic regions during amyloid-β (1–42) fibrillar aggregation and monitored the consequent effects on its aggregation property and structural conversion. Furthermore, we used an amyloid-β (1–42) double point mutant (I41N/A42N) to examine the interactions between the two C-terminal end residues with the two hydrophobic regions and their roles in amyloid self-assembly. Our results indicate that interchain interactions in the central hydrophobic region (^17^LVF^19^) of amyloid-β (1–42) are important for fibrillar aggregation, and its interaction with other domains is associated with the accessibility of the central hydrophobic region for initiating the oligomerization process. Our study provides mechanistic insights into the self-assembly of amyloid-β (1–42) and highlights key structural domains that facilitate this process. Our results can be further applied toward improving the rational design of candidate amyloid-β (1–42) aggregation inhibitors.

## Introduction

Pathogenic amyloid aggregation of intrinsically disordered proteins (IDPs) is related to multiple neurodegenerative diseases.^1^ The pathological hallmark of Alzheimer’s disease (AD)—which is the most prevalent type of dementia—is the accumulation of amyloid-β (Aβ) and microtubule-associated protein (MAP) tau.^2,3^ Fibrillar aggregation of Aβ leads to the formation of senile plaques in the brains of AD patients.^4^ Numerous reports, including genome-wide association studies, imply that the appearance of amyloid aggregates is closely related to neurodegeneration.^5^ Nevertheless, the exact pathophysiological role of the Aβ protein remains unclear.

The Aβ proteins are formed by enzymatic cleavage of the amyloid precursor protein (APP) and commonly have 40 or 42 residues (Aβ40 and Aβ42, respectively).^6^ The longer Aβ42 tends to favor hydrophobic interaction-mediated self-assembly more so in comparison with Aβ40.^7^ The Aβ proteins form various oligomeric species, and each species facilitates the subsequent assembly of several intermediates, eventually culminating in the formation of mature fibrils.^8^ Although multiple studies have presented evidence that the Aβ protein promotes neuronal cell death and hyperphosphorylation of MAP tau, the molecular mechanism of amyloid aggregation is yet to be clearly elucidated owing to the highly heterogeneous nature of the pathological cascade.^9,10^ To date, antibodies and small molecules that selectively bind to aggregates of Aβ fibrils have been introduced as potential therapeutic agents for AD.^11^ In addition, a number of drug candidates target the Aβ-related pathway, which regulates the production or existence of Aβ and modulates the ratio of Aβ proteins.^12^ Although many potential drug candidates against pathogenic amyloid aggregates have been proposed for AD treatment, their effectiveness remains debatable. Therefore, it is necessary to develop a more potent therapeutic strategy for AD based on a more in-depth understanding of the fibrillar amyloid aggregation process at a molecular level.

Recent studies have attempted to rationally design candidate drugs in order to improve existing therapeutic approaches or suppress the fibrillar amyloid aggregation process altogether^13−16^ based on the accumulated knowledge of interlinked dynamics of IDPs, intermediate oligomers, and their pathogenesis to date.^17−20^ For example, a pharmacophore in MAP tau for the binding of small molecules was shown to enhance the binding of potential therapeutic agents.^15^ In addition, an epitope-specific method for antibody design has been reported, which allows development of antibodies to the desired binding epitopes of amyloidogenic IDPs.^16^ Since the self-assembly property of Aβ can be modulated by interrupting or enhancing the contact of the hydrophobic core domains, it would be possible to target the hydrophobic core regions in order to prevent or reduce the capacity of Aβ for self-assembling. Based on such findings, as well as in silico and in vitro analyses, we have previously shown that rationally designed point mutants of Aβ42 can suppress fibrillar aggregation.^14^ In particular, we identified two hydrophobic residues (Phe19 and Ile32) which are crucial for facilitating oligomerization of Aβ42.^14^ The designed point mutants against those sites cross-interacted with Aβ42 in early-stage amyloid aggregation and interfered with the Aβ42 self-assembly.^14^

In this study, we aimed to identify the target region responsible for disrupting fibrillar amyloid aggregation of Aβ42 and further delineate its role in this process. We employed a machine learning (ML) algorithm to predict the binding domain of prefibrillar Aβ42 with various fragments of Aβ42.^21^ Next, we selected two Aβ42 fragments [^16^KLVFFAE^22^, referred to as fragment a (F_a_), and ^25^GSNKGAIIGLM^35^, referred to as fragment b (F_b_), respectively], which displayed self-assembly properties, while also containing one of the two crucial hydrophobic residues (Phe19 and Ile32). Moreover, we designed candidate inhibitor peptides against fibrillar amyloid aggregation of Aβ42 using the two selected fragments and applied a point mutation strategy to eliminate their self-assembly properties. We also confirmed the suppressive effects of the inhibitor candidates on amyloid cluster formation and the alleviation of Aβ42-dependent cytotoxicity in vitro using an SH-SY-5Y cell line. In addition, we examined the disassembly of preformed Aβ42 fibrils upon co-incubation with the inhibitor candidates. To understand the molecular mechanisms underlying the suppression of Aβ42 aggregation, we investigated the interference of inhibitor candidates in early-stage aggregation using an interdisciplinary biophysical approach. Hydrogen–deuterium exchange mass spectrometry (HDX-MS) and ion mobility mass spectrometry (IM-MS) provided evidence of the structural conversion of Aβ42 by designed inhibitor candidates. Combining ML algorithm predictions of peptide-inhibitor complex structures with comprehensive molecular dynamics (MD) simulations provided the molecular basis for peptide-inhibitor interactions. The in silico results and the electron transfer dissociation mass spectrometry (ETD-MS) analysis revealed the binding sites of the inhibitor candidates on Aβ42. We also performed small-angle X-ray scattering (SAXS)-based modeling of the Aβ42 ensembles to understand the solution-phase structural dynamics of Aβ42. Finally, in order to delineate the role of the Aβ42 C-terminal end in amyloid aggregation, we utilized a mutant isoform of Aβ42, I41N/A42N, in which the two C-terminal hydrophobic residues were substituted with polar amino acids.

## Experimental Section

### Predictions of Complex Structures

To predict the complex structures of Aβ42 and its fragments or peptide inhibitor candidates, we employed the AlphaFold multimer (version 2.1.1).^21^ Source code is available at https://github.com/deepmind/alphafold. Structural images were created using UCSF Chimera (version 1.16).^22^

### ThT Fluorescence Assay

Thioflavin T (ThT) assays were used to track the amyloid aggregation of Aβ42 proteins in the presence or absence of the designed peptides. The concentration of Aβ42 was set at 2 and 5 μM. ThT was incubated at 37 °C without agitation in 20 mM Tris–HCl buffer (pH 7.4) in SPL 96-well black microplates (SPL Life Science, Seoul, Korea) with sealing (EASYseal sealing film, Greiner-Bio-One). The designed peptide inhibitor candidates were applied in 1:1, 1:5, or 1:25 molar ratios. Fluorescence intensity was measured continuously at 1 h intervals, for up to 48 h using a Synergy H1 microplate reader (BioTek, Winooski, VT, USA) with excitation and emission wavelengths of 446 and 482 nm, respectively, from the top of the plate. The ThT assays for the I41N/A42N mutant were performed under the same experimental conditions, except for the Aβ42 I41N/A42N concentration (10 μM).

### Transmission Electron Microscopy

Transmission electron microscopy (TEM) images of the Aβ42 fibrils were obtained using negative staining protocols (Supporting Information). Briefly, incubated fibril samples (5 μL) were spotted on a Cu(II) grid for 3 min at 20 °C and removed. The grids were washed twice with HPLC-grade water immediately after sample removal. Then, each sample was stained with 5 μL of uranyl acetate solution (0.5% w/v) for 1 min. The samples were dried for 4 h at 20 °C after removal of the uranyl acetate solution. The TEM images were recorded using a JEM-F200 (TFEG; JEOL Ltd., Japan) field-emission transmission electron microscope [National Center for Inter-University Research Facilities (NCIRF), Seoul National University, Seoul, Republic of Korea] at various magnifications (200 kV; 6000×, 15,000×, 30,000×, and 60,000× magnification).

### Cell Viability Test

MTT assay was used to determine cell viability. In each well of a 96-well plate, SH-SY-5Y cells (15,000 cells) were seeded and incubated for 24 h. Cells were treated with preincubated fibrils in DMEM for 48 h. The MTT solution (5 mg/mL) was added to the medium and incubated for 3 h at 37 °C to form blue MTT-formazan products which were assessed by measuring absorbance at 540 nm using a microplate reader. To ensure reproducibility, each set was tested in triplicate, and the assay was repeated three times.

### Disassembly of Preformed Fibrils

Aβ42 (10 μM) was incubated for 24 h at 37 °C without agitation in 20 mM Tris–HCl buffer (pH 7.4). Fibrillar amyloid aggregation of Aβ42 proteins (10 μM) was monitored using a ThT fluorescence assay. The peptide inhibitor candidates were applied to the preformed fibrils of Aβ42 (diluted to a concentration of 2 μM), and the final peptide inhibitor candidate concentration was 50 μM. Except for the addition of the peptide inhibitor candidate, the control fibrils from the same preformed fibrils of Aβ42 were treated identically. All the samples were incubated at 37 °C without agitation for 24 h. Disassembly of preformed fibrils was monitored by endpoint ThT fluorescence intensity and TEM image analysis at various magnifications (6000×, 15,000×, 30,000×, and 60,000× magnification). The density of the fibrillar aggregates was calculated using the area fraction covered by fibrils in the TEM images. TEM images were further analyzed using the ImageJ software to determine fibril density. Six images at 6000× magnification were processed using MATLAB (version R2020a). The adaptthresh and wiener2 functions were used to implement the adaptive thresholding and noise reduction steps, respectively.

### Small-Angle X-ray Scattering

Solution SAXS experiments were carried out at the 4C SAXS II beamline at the Pohang Accelerator Laboratory (Pohang, Republic of Korea).^23^ All SAXS experiments used freshly prepared samples. Ensemble structures were modeled using EOM analysis from the ATSAS software package (version 3.0.3).^24^ External pools were generated by REMD simulations and used when running a genetic algorithm to optimize ensembles (GAJOE). The ensemble size was set at 50 curves per ensemble, and repetitions were not permitted. The experimental details are described in the Supporting Information.

### MD Simulations

MD simulations and umbrella sampling were performed using the GROMACS software package (version 2020.4).^25^ To estimate the interchain contact probability between the Aβ42 and each peptide inhibitor candidate, a REMD simulation (33 replicas, 150 ns) was performed for each complex system using the CHARMM36m force field and the TIP3P solvent model.^26,27^ The binding free energy was calculated using umbrella sampling combined with a pulling simulation. This was performed using the same force field and solvent model as described above. The 600 ps pulling simulation trajectories were divided into 30 windows, and the MD simulation was run for 10 ns in each window. External generation for EOM analysis was performed using the GROMACS software package (version 4.5.5) with the CHARMM36m force field and the General Born implicit solvation model, where five replicas were simulated for 20 ns.^25,26,28^ The DSSP program was used to calculate the solvent-accessible surface area (SASA) per residue as well as the total hydrophobic or hydrophilic surface area (gmx do_dssp module).

### ESI-MS Combined with the HDX Technique

Freshly prepared Aβ42 and peptide inhibitor candidates were deuterated with D_2_O before quenching with formic acid. The final concentrations of Aβ42 and the peptide inhibitor candidates were 10 and 50 μM, respectively. The samples were immediately injected into a Synapt G2-Si HDMS quadrupole time-of-flight (Q-TOF) mass spectrometer (Waters, Manchester, UK) after the quenching agent was added. The backward-exchanged hydrogens were corrected with D_2_O-hydrated fully deuterated Aβ42.

### Ion Mobility Mass Spectrometry

IM-MS was performed in the positive ion mode on a Synapt G2-Si HDMS Q-TOF mass spectrometer (Waters, UK, Manchester). The final concentration of the Aβ42 protein was 10 μM in 20 mM ammonium acetate (pH 6.8). The designed inhibitors were applied at a concentration of 50 μM, and the proteins were transferred to the gas phase using an electrospray ionization (ESI) source. The ESI parameters and detailed descriptions for calibrating the collision cross-section are given in the Supporting Information.

### Electron Transfer Dissociation Mass Spectrometry

ETD was performed using a Synapt G2-Si HDMS Q-TOF mass spectrometer with ETD functionality (Waters, UK; Manchester). Aβ42 and each peptide inhibitor candidate were mixed at concentrations of 10 and 50 μM, respectively. The samples were introduced at a flow rate of 10 μL/min using an ESI source. To generate anions, the ETD reagent 1,4-dicyanobenzene was introduced into the glow-discharge source. Anions were refilled in the trap cell for 0.2 s at a time interval of 1 s. The ETD mode parameters were a 0.2 V trap wave height, 14 mL/min trap gas flow, and 475 V ETD RF trap. Peak assignment was performed manually by comparing it to a fragment list generated by the ProteinProspector web server (https://prospector.ucsf.edu/prospector/mshome.htm). To compare the MD simulation and ETD-MS results, an interchain contact probability map of the complexes was projected onto the *x*-axis and overlaid on the ETD-MS results.

## Results

### Design of Amyloid Aggregation Inhibitors

**Figure 1:**
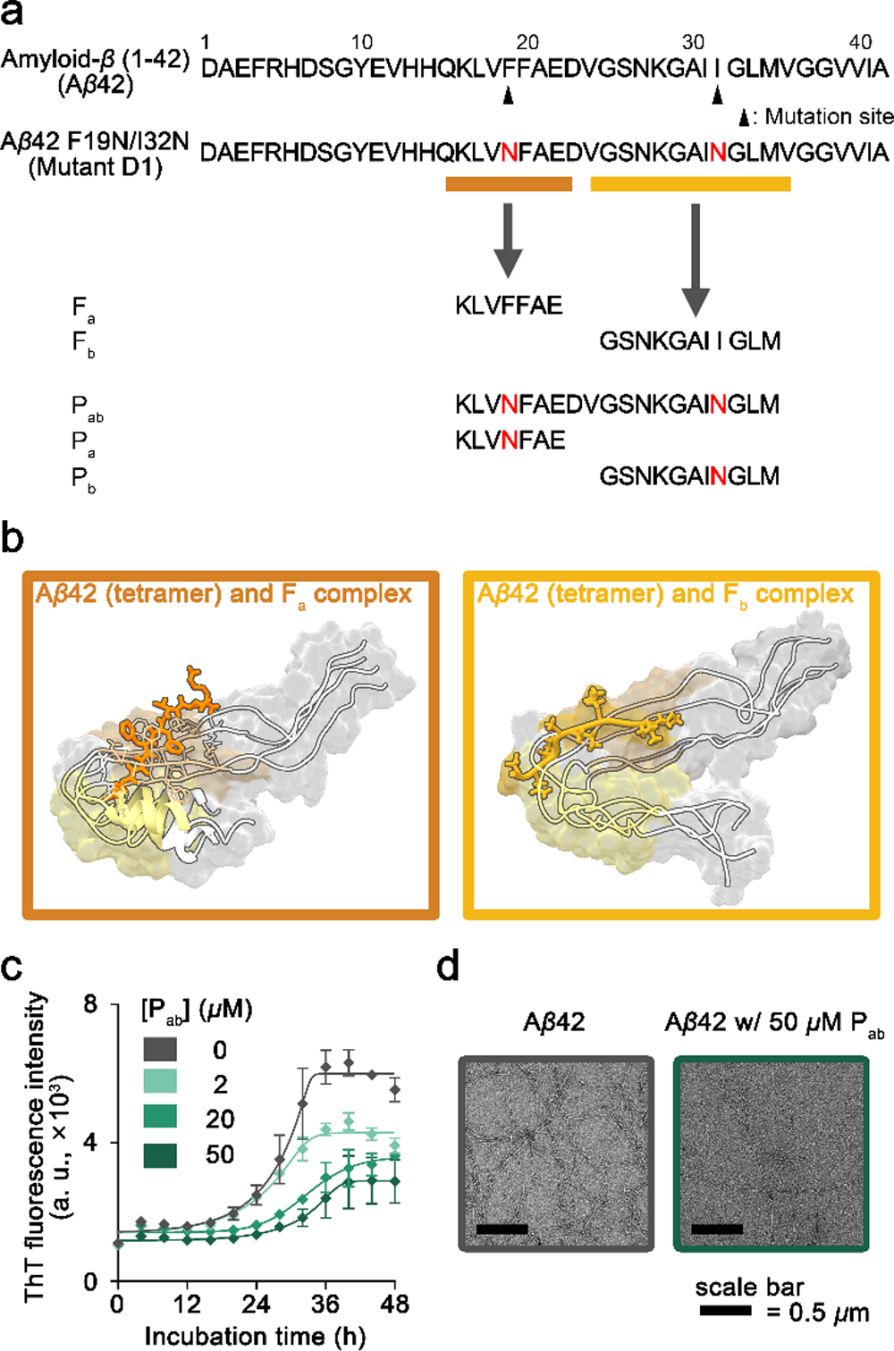
Design of amyloid aggregation inhibitors for amyloid-β (1–42) (Aβ42). (a) Domains of interest are indicated by underlining, and the amino acid sequences of Aβ42 fragments (F_a_ and F_b_) and peptide inhibitor candidates (P_ab_, P_a_, and P_b_) are shown. (b) AlphaFold-predicted structures of the peptide fragment complexes. The Aβ42 tetramer is predicted to stack in parallel, and the fragments (F_a_ and F_b_) are expected to bind to the central hydrophobic region. The central hydrophobic region of Aβ42 (^16^KLVFFAE^22^) is colored as tan, ^25^GSNKGAIIGLM^35^ of Aβ42 as khaki, and each fragment, F_a_ and F_b_, as orange and yellow, respectively. (c) ThT incubation assay for the aggregation kinetics of Aβ42 and mixtures of Aβ42 and peptide inhibitor candidate P_ab_. (d) TEM images of Aβ42 fibrils in Aβ42 and mixtures of Aβ42 and P_ab_. The error bars represent the standard deviation from three independent experiments.

Recent crystallography studies have revealed that the hydrophobic domains of Aβ42 form a hydrophobic core in the fibril structure of Aβ42.^29^ Prompted by previous findings, we designed novel point mutants of Aβ42 to suppress its self-assembly property.^14^ Among the four selected residues (Phe19, Ile32, Leu34, and Ala42), our results showed that Phe19 and Ile32 were essential for fibrillar amyloid aggregation as amino acid substitutions in these positions effectively disrupted the process.^14^ Therefore, we hypothesized that the region including one of the two crucial residues plays a key role in suppressing fibrillar amyloid aggregation of Aβ42. The previously reported mutant D1 (F19N/I32N) cross-interacts with wild-type Aβ42 during early-stage oligomerization and as such interferes with fibrillar amyloid aggregation of Aβ42 (Figure 1a).^14^ Herein, we aimed to identify the key domain which can effectively inhibit Aβ42 aggregation and to target that region by using smaller Aβ42 fragments to modulate the self-assembly function of Aβ42.

Our criteria were that smaller Aβ42 fragments must have self-assembly properties and include at least one of the essential residues (Phe19 or Ile32). To systematically search for binding domains, we compiled previously reported small fragments of Aβ42 (F_a_ to F_q_, 17 fragments in total) and employed AlphaFold to predict the binding of each fragment to prefibrillar Aβ42 (Figures 1b and S1 and Table S1).^30−39^ The Aβ42 tetramer is predicted to stack in parallel, and the fragment peptide KLVFFAE (F_a_) is expected to bind to its cognate region (^16^KLVFFAE^22^) in a parallel conformation. The peptide fragment GSNKGAIIGLM (F_b_) also associates with the central hydrophobic region of Aβ42. However, most of the N-terminal fragments of Aβ42 were expected to bind to the C-terminal end of Aβ42 (Figure S1, F_h_–F_q_). A short fragment, such as IIGLM, also bound to the C-terminal end of Aβ42 (Figure S1, F_g_). Several of the fragments we examined reportedly demonstrated self-assembly properties, and as such, they were expected to bind to the central hydrophobic region.^30,31^ Therefore, we posited that targeting the central hydrophobic region using fragment-based inhibitor candidates could be effective in disrupting the amyloid aggregation process.

Based on the predictions from AlphaFold, we selected two short, aggregate-prone peptides, F_a_ and F_b_, along with their point mutants, to target each domain of Aβ42 with their corresponding sequences while monitoring their effectiveness in inhibiting Aβ42 amyloid aggregation (Figure 1a). The peptides F_a_ and F_b_ both include the mutation target sites mentioned above and are also specific for the central and C-terminal regions, respectively. These two peptides have been reported to form insoluble amyloid bodies in vitro and are widely used as model systems in amyloid aggregation studies.^30,31^ We monitored the cluster formation of the two fragments using a ThT fluorescence assay, whose fluorescence intensity increases upon binding to amyloid fibrils, and found that both fragments have self-assembly capabilities (Figure S2). As the intermolecular contacts are mainly initiated by hydrophobic interactions, we expected that each fragment with self-assembly properties would still be able to cross-interact with Aβ42 via the corresponding domain.

We chose KLVNFAEDVGSNKGAINGLM (P_ab_) as our first experimental inhibitor candidate peptide because it spans both the^16^KLVFFAE^22^ (F_a_) and ^25^GSNKGAIIGLM^35^ (F_b_) regions and contains both F19N and I32N point mutations (Figure 1a). The suppressive effect of the designed peptide inhibitor candidate P_ab_ on Aβ42 amyloid aggregation was monitored by a ThT incubation assay and TEM image analysis after negative staining (Figure 1c,d). The Aβ42 sample showed an increase in the ThT fluorescence intensity as a function of time. The mixture of Aβ42 and P_ab_ at equimolar concentrations displayed lower ThT fluorescence intensity than the Aβ42 sample at plateau intensity. In addition, the peptide inhibitor candidate P_ab_ suppressed fibrillar amyloid aggregation in a concentration-dependent manner (Figure 1c). The TEM image analysis supports our ThT assay results, showing fewer amyloid fibrils in the mixture of Aβ42 and P_ab_ (Figure 1d).

### Effects of Fragments and Designed Peptides on Fibrillar Amyloid Aggregation of Aβ42

**Figure 2:**
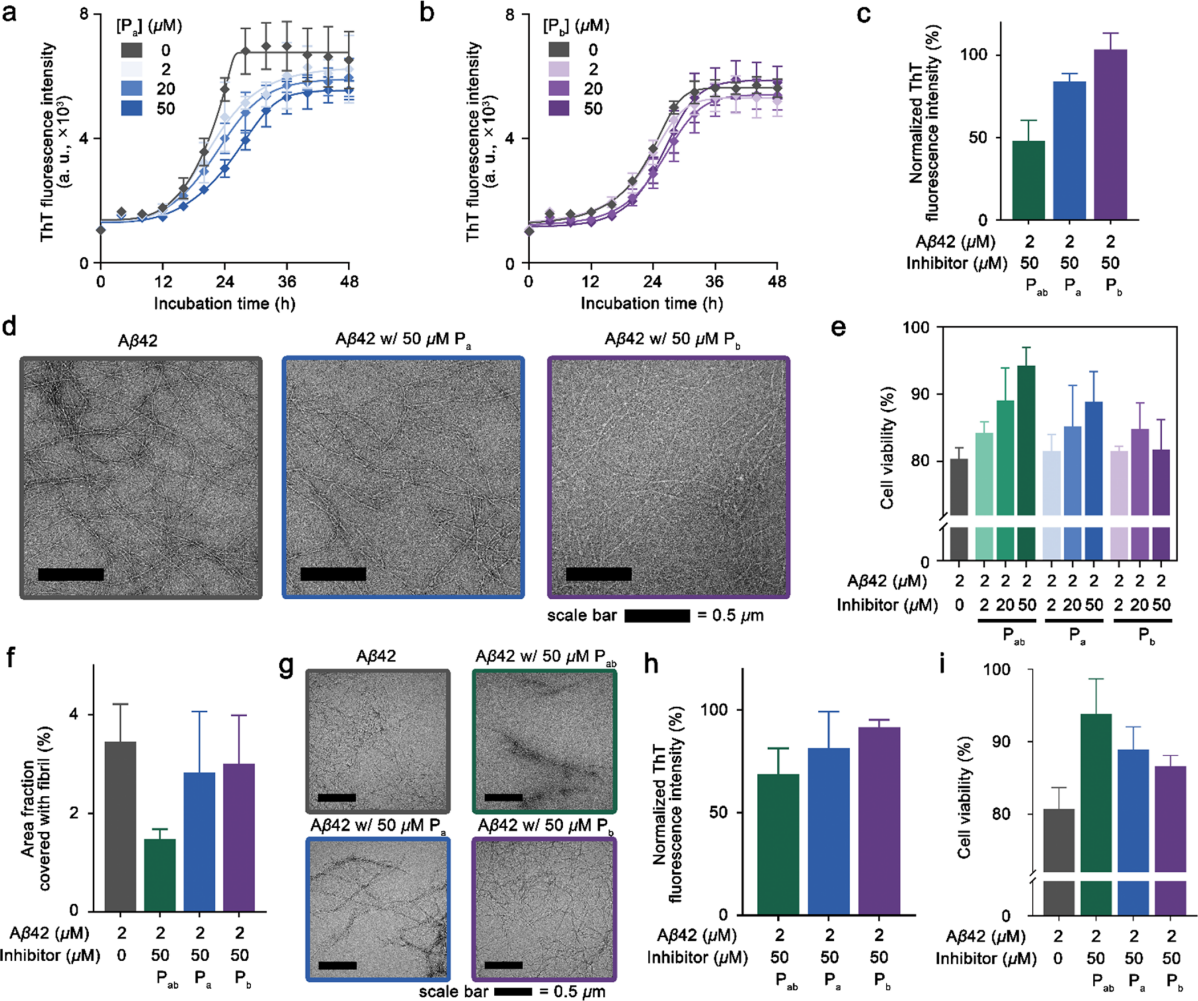
Effects of peptides and inhibitor candidates on fibrillar amyloid aggregation of Aβ42. ThT fluorescence assays were used to monitor the aggregation kinetics of the mixture of Aβ42 and (a) P_a_ or (b) P_b_. (c) Normalized ThT fluorescence intensity of Aβ42 and the mixture of Aβ42 with inhibitor candidates after 48 h incubation. (d) TEM images of Aβ42 fibrils in mixtures of Aβ42 and peptide inhibitor candidates. (e) MTT assay to monitor the cytotoxicity of Aβ42 and mixtures of Aβ42 with the three designed mutant candidates. The samples were preincubated for 48 h and added to the SH-SY-5Y cells, followed by an additional 48 h incubation. (f, g) Disassembly of preformed Aβ42 fibrils monitored by TEM image analysis. Designed inhibitor candidates added to preformed fibrils and incubated 24 h at 37 °C. (h) Normalized ThT fluorescence intensity at the endpoint of the disassembly assay. (i) MTT assay to monitor the cytotoxicity of preformed Aβ42 fibrils after inhibitor-induced disassembly. The error bars represent the standard deviation from three independent experiments, except for fibril density analysis (standard deviation from six images).

To understand the specific effects of different regions on fibrillar amyloid aggregation, we further examined how the aggregation of Aβ42 is affected by two smaller peptide inhibitor candidates, KLVNFAE (P_a_) and GSNKGAINGLM (P_b_), which span the central and C-terminal hydrophobic regions, respectively. While both P_a_ and P_ab_ suppressed Aβ42 aggregation to an extent, P_ab_ was more effective in this regard than P_a_ (Figures 1c and 2a,c). Interestingly, the inhibitor candidate P_b_ did not suppress Aβ42 aggregation (Figure 2b,c). The TEM image analysis agreed with the ThT assay results, suggesting an inhibitory effect of P_a_ on Aβ42 aggregation, while no considerable effect was observed for P_b_. Notably, both P_ab_ and P_a_, which contain the F19N point mutation, effectively suppressed Aβ42 amyloid aggregation (Figures 1d and 2d). We further investigated the inhibitory effect of designed peptides on Aβ42 aggregation at higher Aβ42 concentration (25 μM) since distinct forms of the Aβ oligomer have been reported above the critical concentration (20–25 μM).^40,41^ The results show that even though the Aβ42 concentration increased, the suppressive effect of the designed peptides persisted (Figure S6).

To monitor the cellular responses after addition of the designed inhibitor candidates, we performed the cytotoxicity test on the SH-SY-5Y cells using the modified thiazolyl blue tetrazolium bromide (MTT) (Figure 2e). We incubated the pure Aβ42 sample, as well as mixtures of Aβ42 and the designed peptides for 48 h at 37 °C, and applied these to SH-SY-5Y cells. The results showed that the cytotoxicity of Aβ42 decreased significantly as the concentration of P_ab_ increased. The inhibitor candidate P_a_ also alleviated the cytotoxicity of Aβ42 in a concentration-dependent manner. In contrast, P_b_ did not significantly diminish the cytotoxicity of Aβ42. These findings indicated that suppression of Aβ42 aggregation by the designed peptide inhibitors, P_ab_ and P_a_, also reduced the toxicity of Aβ42 (Figures 1c and 2a,e).

Moreover, we assessed the disassembly of amyloid Aβ42 clusters using these three peptides. After a 24 h incubation period, we applied the designed peptides to the preformed Aβ42 fibrils and allowed these to act for a further 24 h at 37 °C. The TEM image analysis revealed that the designed peptides affected the density of the fibrillar aggregates (Figure 2f,g). The area fraction covered with fibrils in the TEM images was used to calculate the density of fibrillar aggregates. The density of fibrillar aggregates in the TEM images of the Aβ42 sample was 3.45%, and this decreased to 1.48, 2.82, and 3.01% upon addition of P_ab_, P_a_, and P_b_, respectively (Figure 2f). The endpoint ThT fluorescence intensity of each sample was monitored to quantify ThT-positive aggregates. Compared to the Aβ42 sample treated with incubation buffer (as a negative control), the ThT fluorescence intensity in each of the Aβ42-inhibitor mixture samples treated with either P_ab_, P_a_, and P_b_ decreased to 68, 81, and 92%, respectively (Figure 2h). The designed peptide P_ab_, which covered both the F_a_ and F_b_ regions in Aβ42 and included F19N and I32N point mutations, induced the disassembly of preformed amyloid aggregates of Aβ42. The P_a_ designed peptide, which covers the F_a_ region in Aβ42 and includes an F19N point mutation, seemed to have similar trends to P_ab_. However, P_b_, the sequence of which corresponds to that of F_b_ and contains an I32N point mutation, did not show notable disassembly of the preformed amyloid aggregates of Aβ42. The MTT assay showed alleviated cytotoxicity of preformed Aβ42 fibrils after inhibitor-induced disassembly, and the results were in good agreement with our fibril disassembly assays (Figure 2i).

### Identification of the Three Key Domains of Aβ42

**Figure 3:**
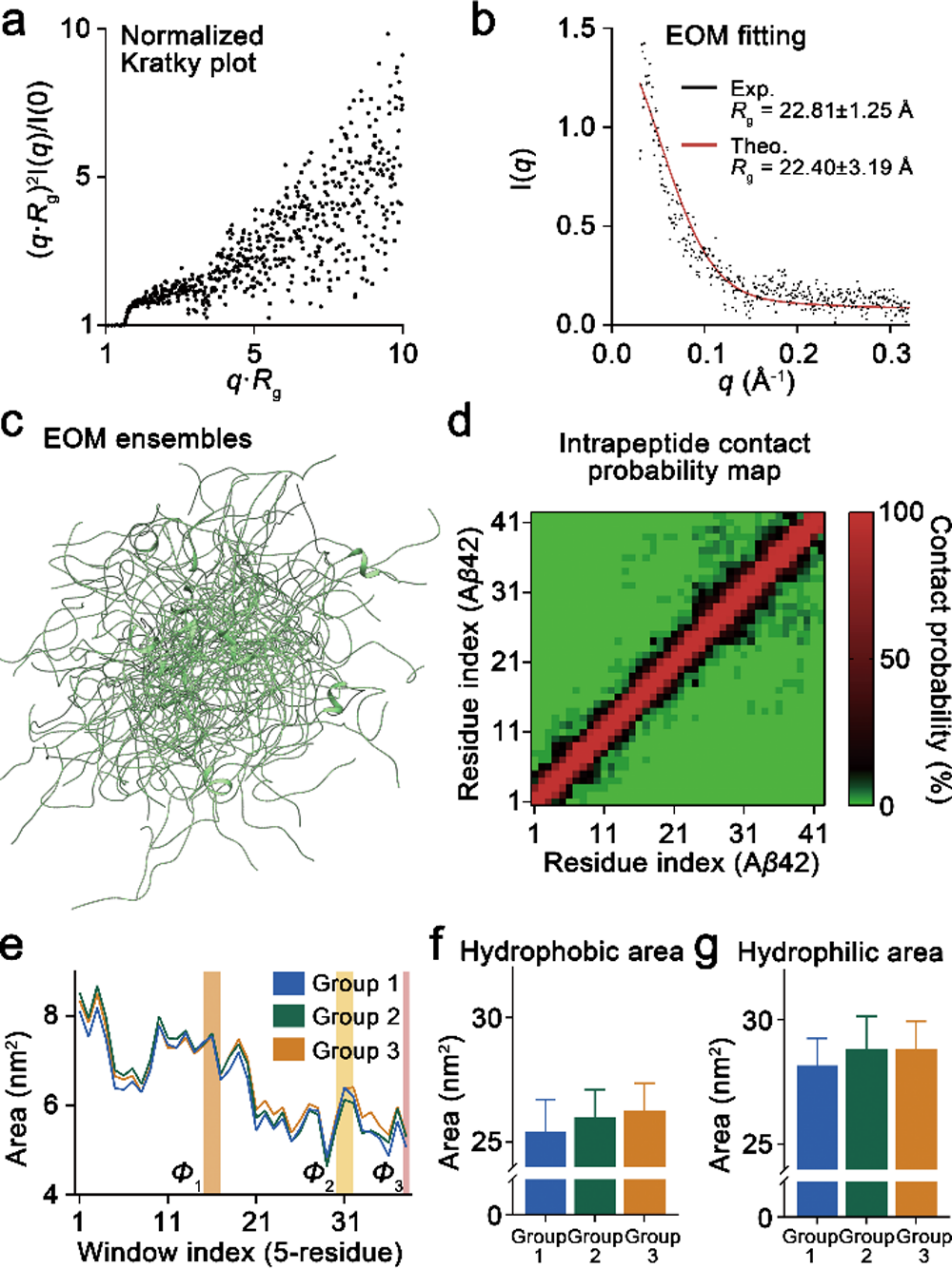
Modeling of solution-phase amyloid-β (1–42) (Aβ42) ensembles using SAXS. (a) Normalized Kratky plot of Aβ42 based on the SAXS profile. (b) EOM analysis of the raw SAXS profile of Aβ42 (black) and the fitted curve (red). (c) Ensemble structures of Aβ42 obtained using EOM analysis. (d) EOM-derived intrapeptide contact probability map of the Aβ42 ensembles. (e) 50 EOM structures are divided into three groups by the radius of gyration, and the SASA of the adjacent five residues is plotted. Window 1 means residues 1–5, and window 2 means residues 2–6. (f, g) Averaged SASA calculated from three groups of EOM structures with (f) hydrophobic and (g) hydrophilic areas. The error bars represent the standard deviation.

To understand the different roles of the three regions (central hydrophobic region, ^17^LVF^19^, Φ_1_; C-terminal hydrophobic region, ^32^IGL^34^, Φ_2_; and C-terminal end, ^41^IA^42^, Φ_3_) (Figure S7), we investigated the solution-phase dynamics of Aβ42 using SAXS experiments. We performed an ensemble optimization method (EOM) analysis since the Kratky plot showed that Aβ42 has a highly unstructured conformation (Figure 3a), and the theoretical analysis was well fitted to the experimental curve (*R*_g,theo_ = 22.40 ± 3.19 Å and *R*_g, exp_ = 22.81 ± 1.25 Å, respectively) (Figure 3b). Fifty ensemble structures of Aβ42 were obtained from EOM analysis (Figure 3c) and subjected to calculations for the intramolecular contacts of each structure. The resulting intramolecular distances were plotted as a contact probability map of Aβ42 to better understand the transient interactions in the monomeric state of Aβ42 (Figure 3d). We observed that the Φ_2_ Aβ42 site interacts with both Φ_1_ and Φ_3_ on the same molecule. The interaction between Φ_2_ and Φ_3_ should be avoided for the successful association of Φ_1_ with Φ_2_. The latter interaction shields the Φ_1_ domain by positioning it inward in the Aβ42 structure. Based on these observations, we speculate that the structural fluctuations of Aβ42 originate from intramolecular hydrophobic interactions in the C-terminal regions.

We separated the modeled ensembles into three groups according to the radius of gyration (compact conformers were assigned to group 1, intermediates to group 2, and extended conformers to group 3) to deconvolute the structural basis of the Aβ42 fluctuations (Figure S8). We assessed the exposure of each domain (5-residue windows) based on SASA (Figure 3e). These results suggest that Φ_2_ is mostly located in the interior sites of the protein; thus, Φ_1_ is likely exposed to solvent molecules and other Aβ42 peptides. The total solvent-accessible hydrophobic surface area decreased in proportion to the radius of the Aβ42 gyration (Figure 3f). The sum of the solvent-accessible hydrophilic surface area also decreased in group 1, which suggests that compact conformations of Aβ42 lead to the shielding of both hydrophobic and hydrophilic surface areas (Figure 3f,g). On the other hand, the intermediate conformers of Aβ42 did not induce shielding of the hydrophilic surface area, which indicates that the structural fluctuations of Aβ42 conformers are mainly driven by intramolecular contacts of the hydrophobic regions (Figure 3g). Investigation of the solution-phase dynamics of Aβ42 revealed the interactions between its three key domains (Φ_1_, Φ_2_, and Φ_3_). These observations showcase that the deviation in the solvent-accessible area between groups 2 and 3 mainly arises from two intramolecular hydrophobic interactions: between Φ_1_ and Φ_2_ or between Φ_2_ and Φ_3_ (Figure 3e–g). We previously reported that self-assembly of Aβ42 initiates with the interchain hydrophobic contacts of Φ_1_ and Φ_2_.^14^ Intramolecular hydrophobic interaction between Φ_1_ and Φ_2_ leads to shielding of both interchain interaction sites. However, when Φ_2_ interacts with Φ_3_, the Φ_1_ domain is consequently exposed. Based on these findings, we hypothesized that Φ_1_ is shielded by Φ_2_, thereby delaying fibrillar amyloid aggregation. As such, it may be possible that the shielding effect of Φ_2_ could be weakened when the domain interacts with Φ_3_.

### Molecular Mechanisms Underlying Suppressed Amyloid Aggregation of Aβ42

**Figure 4:**
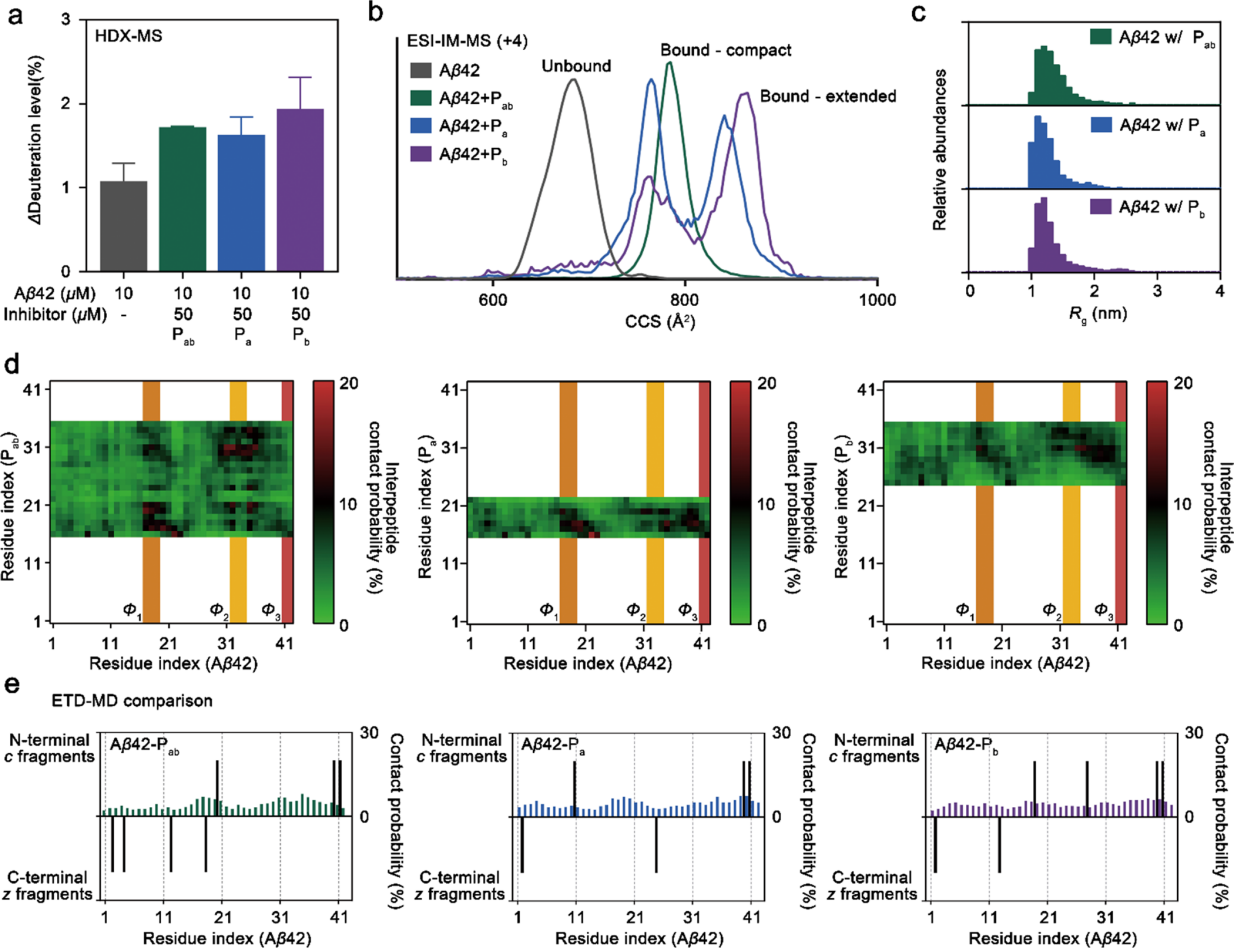
Investigation of the structural dynamics of the amyloid-β (1–42) (Aβ42) after the addition of the three designed inhibitor candidates. (a) Increment in the normalized deuteration level for Aβ42 with and without the addition of the designed inhibitor candidates. (b) Distributions of experimental collision cross-section (Ω_D_) of Aβ42 and Aβ42-inhibitor complexes. (c) *R*_g_ distributions of the Aβ42 conformers obtained from the REMD simulations of Aβ42-inhibitor complexes. (d) Interpeptide contact probability maps of the three Aβ42-inhibitor complexes from 24.75 μs MD simulations. (e) Comparison of the residue-specific interpeptide contact probabilities obtained from MD simulations and bindings between Aβ42 and inhibitor candidates, which were monitored by using ETD-MS fragmentation of the Aβ42-inhibitor complex. The product ions are indicated in gray, and the MD simulated contact probability is represented in green, blue, and purple (Aβ42-P_ab_, Aβ42-P_a_, and Aβ42-P_b_, respectively). The error bars represent the standard deviation from three independent experiments.

We performed MS-based structural analyses to further investigate the molecular mechanisms underlying the suppression of the fibrillar amyloid aggregation process. The deuteration level of proteins during HDX reflects the accessibility of protein regions to solvent molecules. A higher HDX propensity indicated more frequent solvent exposure with an extended conformation. We employed HDX-MS analysis to monitor the structural conversion of Aβ42 in the presence of the designed peptides (Figure 4a). As a reference, the increment in the deuteration level of inhibitor-free Aβ42 (incubated for 1 s in water prior to acidic quenching) during the MS data acquisition for 90 s was 1.07 ± 0.21%. All three designed inhibitor candidates facilitate Aβ42 deuteration, with P_b_ causing the greatest increase in Aβ42 deuteration level (1.72 ± 0.01, 1.63 ± 0.21, and 1.94 ± 0.38% for P_ab_, P_a_, and P_b_, respectively). Therefore, it is anticipated that the designed peptides, in general, induce Aβ42 to be extended by peptide–protein interactions.

Next, we attempted to dissect the interaction modes of the Aβ42 complexes with different peptides. The structural dynamics of Aβ42 and Aβ42-inhibitor complexes were monitored in the gas phase using IM-MS by determining the collision cross-sections (CCSs) of each complex ion (Figure 4b). According to the IM spectra, the complex ions (+4 charge state) showed increased CCS values compared to free Aβ42 ions. The complex ions of Aβ42 with P_a_ or P_b_ showed two conformers (compact and extended conformers), while the Aβ42 complex ion with P_ab_ did not show extended conformers (Figure 4b). It should be noted that the compact conformer of the Aβ42–inhibitor complex ion is still extended compared to the intact Aβ42 ion’s conformer (Figure 4a,b). The Aβ42-P_a_ complex ions preferred a compact conformation over the extended conformation. In contrast, the P_b_-bound complex ions displayed an increase in the population of extended conformers. Taken together, the Aβ42-P_ab_ complex ions presented only with a compact conformer, while Aβ42 complexes with the other designed peptides showed both compact and extended conformers. In addition, the Aβ42 complex ions with P_a_ and P_b_ showed extended conformers to a significant extent. The Aβ42-P_b_ complex ions displayed a preferential tendency for extended conformers in contrast to the Aβ42-P_a_ complex. The degree of extension of Aβ42 may play an important role in suppressing the fibrillar amyloid aggregation of Aβ42.

To further investigate the atomistic details of the observed structural changes in Aβ42 and various amyloid complexes, we utilized AlphaFold to predict complex structures between the Aβ42 monomer and each of the three designed peptides. The major binding site was the central hydrophobic region of Aβ42 (Figure S11). The complex structures were fed into replica-exchange MD (REMD) simulations as initial structures. The REMD simulation sets consisted of 33 different temperatures ranging from 300 to 400 K with equal exchange probabilities. For each designed peptide, we obtained five *R*_g_ distributions of Aβ42 and five interpeptide contact probability maps from independent REMD simulations and weight-averaged them based on the binding free energy obtained by umbrella sampling (Table S2). The *R*_g_ distributions of Aβ42 when the protein coexists with the designed peptide P_b_ showed an extra population with an extended conformation centered at 2.4 nm (Figure 4c). The HDX-MS results and *R*_g_ distribution data indicate that P_b_ induces Aβ42 to be extended more than the other two designed peptides (Figures 4a,c). All designed peptides bound to Aβ42 without a specific interaction site (Figure 4d). We observed a preference for peptide inhibitors associating with the central hydrophobic region along with the C-terminal hydrophobic region of Aβ42. P_ab_, which spans both the two hydrophobic regions of Aβ42, interacted with Φ_1_ and Φ_2_ of Aβ42 as expected. P_a_ and P_b_, whose sequences correspond to the Φ_1_ and Φ_2_ regions on Aβ42, respectively, also associated with Φ_1_. It is noteworthy that both P_a_ and P_b_ interacted with the Aβ42 Φ_3_ domain. Although P_a_ and P_b_ were designed to bind only to their respective Φ_1_ and Φ_2_ cognate sites on Aβ42, we observed that these inhibitors could also concomitantly interact with the Φ_1_ and Φ_3_ domains of Aβ42. Because P_a_ preferred contact with the Φ_1_ domain of Aβ42 over the Φ_3_ region, P_a_ showed effective suppression of Aβ42 aggregation (Figure S13). On the other hand, P_b_ still contacts the Φ_1_ domain most frequently, but contact probabilities do not differ significantly among the three hydrophobic domains (Figure S13). P_ab_, which suppressed Aβ42 aggregation the most effectively, also made significant contacts with the Φ_2_ domain of Aβ42, but P_ab_’s binding to the Φ_1_ and Φ_2_ domains occurs concomitantly not independently (Figure S14). The transient binding of designed peptides into the Φ_3_ of Aβ42 may disrupt intramolecular contact between Φ_2_ and Φ_3_ (Figure S15).

We experimentally examined the probable binding sites of Aβ42 peptide inhibitors by ETD analysis. We used 1,4-dicyanobenzene to generate anions, and the chemical reaction between the Aβ42 cation and radical reagent anion led to peptide backbone cleavage. The ETD-MS results revealed that the peptide inhibitors were located around multiple regions of Aβ42 (Figure 4e). Structural analysis of Aβ42 and inhibitor complexes suggests that P_a_ and P_b_ could interact with Aβ42 in two different hydrophobic regions (Φ_1_ and Φ_3_, respectively). Although we found that P_a_ and P_b_ could each separately occupy the Φ_1_ and Φ_3_ Aβ42 sites at the same time, the binding of the designed peptides to different regions may lead to structural conversion of Aβ42 to different extents. Taken together, interaction of the designed peptides with the specific hydrophobic region of Aβ42 leads to conformational changes and suppresses protein aggregation.

In summary, the designed peptides interacted with Aβ42 in multiple hydrophobic domains. The P_ab_ inhibitor associated with the Φ_1_ and Φ_2_ regions of Aβ42, whereas the other peptides interacted with Φ_1_ and Φ_3_. Since binding of either P_ab_ or P_a_ leads to the extension of Aβ42 to a similar degree and conveys an inhibitory effect on fibrillar amyloid aggregation, we are prompted to conclude that the Φ_1_ domain on Aβ42 is likely the main target for suppression of amyloid aggregation (Figures 1c, 2a, and 4). These results also demonstrate that binding of inhibitor candidates to the C-terminus of Aβ42 results in an extension of the Aβ42 conformation and cannot effectively disrupt fibrillar amyloid aggregation (Figures 2b and 4). The AlphaFold-predicted binding sites of F_a_ and F_b_ in prefibrillar Aβ42 are also the central hydrophobic regions of the protein. This implies a potential competition for interactions near the central hydrophobic region, where they may also bind adjacent Aβ42 molecules in the amyloid fibrils, inducing Aβ42 fibril disassembly (Figure 1b).^15^

### Examination of the Role of Three Key Domains in Aβ42 Self-Assembly

Multiple lines of experimental evidence have indicated that Φ_1_ is the main target for inhibition of fibrillar amyloid aggregation. Based on the SAXS experiment, we hypothesized that Φ_1_ would be shielded by Φ_2_, thereby delaying fibrillar amyloid aggregation, whereas this shielding effect would be weakened when the Φ_2_ domain interacted with Φ_3_. To test our hypothesis, we used a double point mutant of Aβ42 (I41N/A42N) in order to disrupt the association between the Φ_2_ and Φ_3_ regions on Aβ42. It is commonly accepted that the two hydrophobic residues at the C-terminal end of Aβ42 enhance the initiation of the fibrillar amyloid aggregation process compared to Aβ40.^7^ The ThT fluorescence assay was performed to compare the fibrillation kinetics between Aβ42 and the double point mutant isoform (Aβ42 I41N/A42N) in the same peptide concentration (Figure S17). We observed that the point mutations caused a significant kinetical delay in fibrillar amyloid aggregation (*t*_1/2,Aβ42_ = 11.37 ± 0.82 h and *t*_1/2,Aβ42I41N/A42N_ = 22.07 ± 1.69 h).

**Figure 5:**
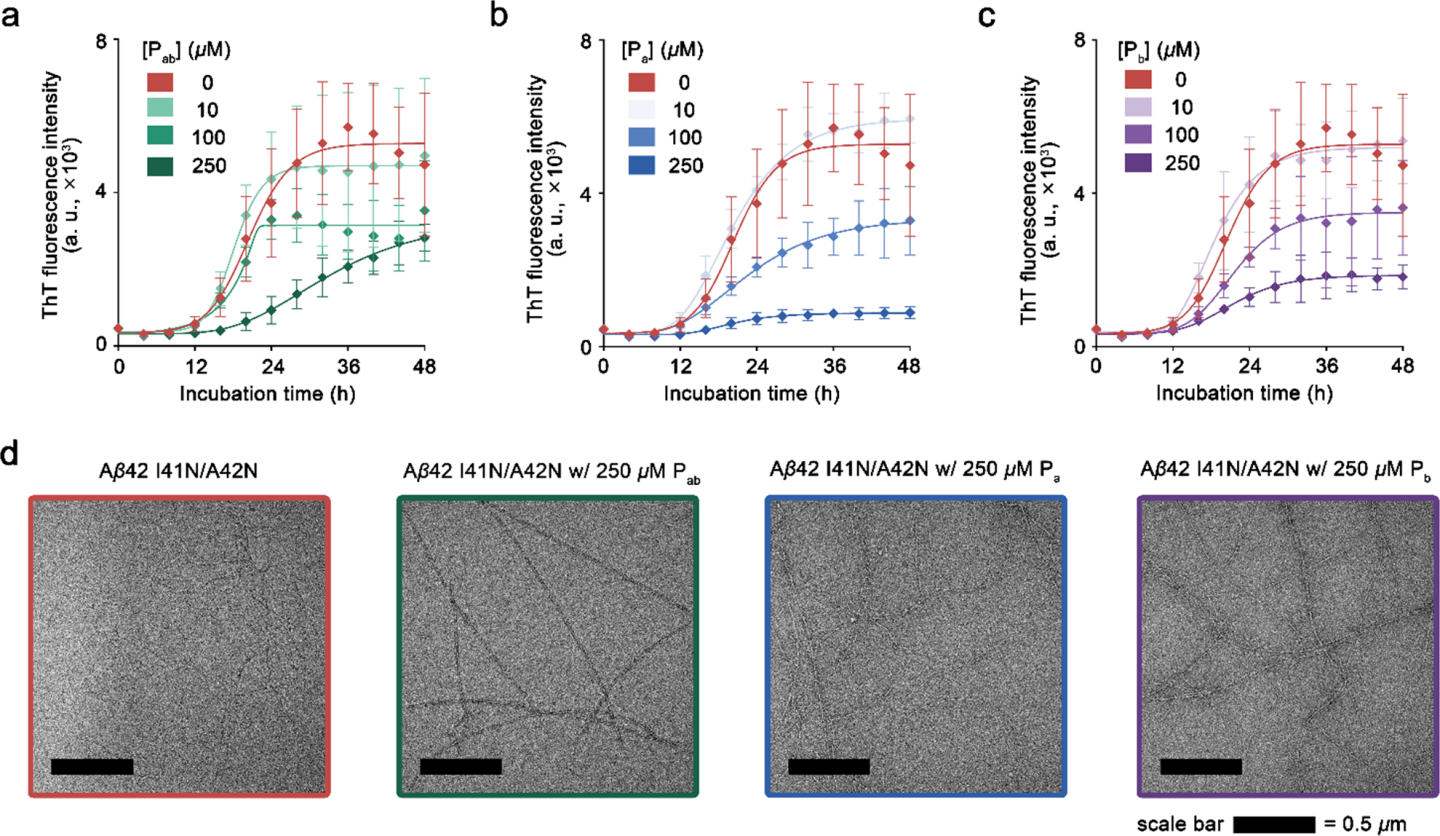
Comparison of amyloid aggregation in mixtures of amyloid-β (1–42) I41N/A42N point mutant and three designed inhibitor candidates. (a–c) ThT fluorescence assay was used to monitor the amyloid aggregation of (a) amyloid-β (1–42) I41N/A42N and mixtures of amyloid-β (1–42) I41N/A42N and P_ab_, (b) mixtures of amyloid-β (1–42) I41N/A42N and P_a_, and (c) mixtures of amyloid-β (1–42) I41N/A42N and P_b_. (d) TEM images of mature fibrils of amyloid-β (1–42) I41N/A42N with and without the designed inhibitor candidates. The error bars represent the standard deviation from three independent experiments.

Moreover, we revealed that the hydrophobic interaction-driven binding of the designed peptides (P_a_ and P_b_) was separated into multiple sites, including Φ_1_ and Φ_3_ (Figure 4d,e). To investigate the effects of focusing the binding of designed peptides into Φ_1_ and eliminating competition for this domain, we applied the designed inhibitors to the Aβ42 I41N/A42N mutant. We traced the aggregation kinetics of mixtures of the double point mutant (Aβ42 I41N/A42N) and three designed inhibitor candidates. P_ab_ and P_a_ still had an inhibitory effect on fibrillar amyloid aggregation in a concentration-dependent manner (Figure 5a,b). In addition, P_b_, which failed to abolish the fibrillar amyloid aggregation of wild-type Aβ42, did suppress the process effectively in the mutant isoform (Figure 5c). The TEM image analysis results were in close agreement with those of the ThT incubation assay (Figure 5d).

Interestingly, all three inhibitor candidates showed inhibitory effects to a similar degree, in contrast to wild-type Aβ42 amyloid aggregation (Figures 1 and 2). We confirmed that the conformations of the complex ion (Aβ42 I41N/A42N-P_b_) merged into a single distribution in the gas phase of the IM spectra (Figure S18). These findings indicate that focusing the binding of designed peptides to Φ_1_ suppresses fibrillar amyloid aggregation, highlighting Φ_1_ as the primary target for suppressing Aβ42 aggregation. Our previous study, which reported the structural difference between Aβ40 and Aβ42, also suggests that absence of the C-terminal end enhances the interaction between the central hydrophobic and the C-terminal hydrophobic regions, thereby increasing the population of compact conformers of Aβ40 compared to Aβ42.^42^ Since the central hydrophobic region has been reported to be the key region for initiating Aβ40 aggregation as well, the limited role of the C-terminal end of Aβ42 on fibrillar amyloid aggregation was observed in comparison with that of both Aβ42 I41N/A42N and Aβ40.^43,44^

## Conclusions

In this work, we investigated the roles of the central (^17^LVF^19^, Φ_1_) and C-terminal hydrophobic (^32^IGL^34^, Φ_2_) regions, as well as the C-terminal end (^41^IA^42^, Φ_3_) of Aβ42 in the fibrillar amyloid aggregation process, using Aβ42 fragments. Taken together, our computational and experimental data suggest that: (1) the Φ_1_ domain drives Aβ42 amyloid aggregation and that (2) the Φ_2_ Aβ42 site can interact with either Φ_1_ or Φ_3_. If Φ_3_ is absent or is replaced by hydrophilic residues, then Φ_2_ constantly (yet transiently) interacts with Φ_1_, which disrupts early-stage oligomerization. When Φ_3_ is involved in hydrophobic interactions, this preferentially binds to Φ_2_, in turn increasing the likelihood of the Φ_1_ domain being more exposed to solvent molecules. This exposure can lead to intermolecular hydrophobic interactions, which drive the oligomerization of Aβ42.

Our HDX-MS experiments revealed that nonspecific binding of designed peptides to Aβ42 induces the conversion of the Aβ42 structure to a more extended conformation, which is related to a lack of intramolecular hydrophobic interactions. Based on structural analyses of Aβ42 complexes using IM-MS, REMD simulations, and ETD-MS, we also observed that binding of the designed peptides to the Φ_1_ domain of Aβ42 suppresses fibrillar Aβ42 aggregation. This compelling evidence suggests that the designed peptides’ association with the Φ_1_ domain of Aβ42 can disrupt intermolecular interactions, thereby interfering with Aβ42 cluster formation. Furthermore, we identified intramolecular interactions of Aβ42 using SAXS and MD simulations. The structural analysis results indicated that intramolecular hydrophobic interactions of the Φ_2_ Aβ42 site determine the monomeric Aβ42 conformation and accessibility to the Φ_1_ domain of the protein. To assess the consequent implications on Aβ42 aggregation, we monitored the effects of substituting the C-terminal end with a polar amino acid using the Aβ42 I41N/A42N mutant and focusing the intramolecular interactions of Φ_2_ to the Φ_1_ domain. Our findings revealed that the aggregation kinetics of the double point mutant were delayed compared to that of wild-type Aβ42.

Because the Aβ42 I41N/A42N mutant and Aβ40 share similar features during the kinetical delay of fibrillar amyloid aggregation, we suggest that the probable role of the Aβ42 C-terminal end is to interfere with the interaction between Φ_1_ and Φ_2_. We also observed the binding of the designed inhibitor candidates to Aβ42 by combining the MD simulations with MS-based analysis. Multiple lines of evidence indicate that disruption of interpeptide interactions between the Φ_1_ domains suppresses the self-assembly properties of Aβ42. The Φ_2_ Aβ42 site is not involved in catalyzing the formation of early-stage oligomers but, conversely, slows down the fibrillar amyloid aggregation process of Aβ42. We previously reported that the double point mutation I32N/L34N of Aβ42 promotes the self-assembly of the protein.^14^

Based on our findings, we designed potential inhibitors of Aβ42 amyloid aggregation. Our hypothesis was simple: if the inhibitor reduces the likelihood of the Φ_1_ domain being exposed, then oligomerization (and fibrillation) may be hampered. The key hydrophobic residues (F19 and I32) of the Aβ42 fragments were replaced by asparagine, which disrupted the alignment of the different Aβ42 chains. The designed peptides, P_ab_ and P_a_, both of which spanned the Φ_1_ domain, could suppress fibrillar amyloid aggregation while alleviating the cytotoxicity of Aβ42 and promoting the disassembly of preformed fibrils. In contrast, another designed peptide, P_b_, which lacks the Φ_1_ domain, failed to disrupt the self-assembly properties of Aβ42.

A higher preference for interacting with the Φ_1_ domain leads to the effective suppression of fibrillar amyloid aggregation in the context of drug discovery. Our findings herein prompt us to suggest that the seven-residue peptide, P_a_, could be a viable candidate as a therapeutic agent that may be further tested in preclinical models. Furthermore, this peptide inhibitor could also be potentially utilized in drug development as an adjuvant to existing therapeutic strategies, such as antibodies and molecular receptors, in order to increase biocompatibility, improve delivery techniques, and enhance the target molecule’s binding affinity for the Φ_1_ domain of Aβ42. Although binding of P_a_ to the Φ_1_ domain of Aβ42 suppresses protein aggregation, it may interact with Aβ42 in multiple domains competitively. According to our findings, competitive binding reduces the efficacy of P_a_; thus, combining it with other approaches that increase selectivity to the Φ_1_ domain of Aβ42 would be helpful to overcome its limitations.

Our proposed target domain may additionally be used in other types of drug design, including small-molecule inhibitors. Overall, our approaches for investigating inter- and intramolecular interactions and binding with multiple IDP binding sites may be utilized in the development of alternative candidate drugs against several amyloid-deposition neuropathology.

## Supporting Information Available

Additional experimental details, materials, and methods, including photographs of experimental setup (PDF)
