# Supplementary material for: Decoding the Roles of Amyloid-β (1–42)’s Key Oligomerization Domains toward Designing Epitope-Specific Aggregation Inhibitors
Source: JACS Au. 2023 Mar 2;3(4):1065–75. doi: 10.1021/jacsau.2c00668 (PMC10131210; doi:10.1021/jacsau.2c00668)
Supplement: Supplementary file 1 — au2c00668_si_001.pdf [file au2c00668_si_001.pdf]

Supplemental Information

Decoding the Roles of Amyloid-β (1-42)'s Key Oligomerization Domains Toward Designing Epitope-Specific Aggregation Inhibitors

Dongjoon Im[†,‡,§], Soohyeong Kim [†,‡,§], Gyusub Yoon [†,‡,§], Da Gyeong Hyun [†,‡,§], Yu-gon Eom [¶], Ye Eun Lee[†], Chang Ho Sohn [|, ‖], Jeong-Mo Choi [*,¶,⊥], and Hugh I. Kim [*,†,‡,§]

[†]*Department of Chemistry, Korea University, Seoul 02841, Republic of Korea*

[‡]*Center for Proteogenome Research, Korea University, Seoul 02841, Republic of Korea*

[§]*Single Cell Analysis Laboratory, Korea University, Seoul 02841, Republic of Korea*

[¶]*Department of Chemistry, Pusan National University, Busan 46241, Republic of Korea*

[|]*Center for Nanomedicine, Institute for Basic Science (IBS), Seoul 03722, Republic of Korea*

[‖]*Graduate Program in Nanobiomedical Engineering, Advanced Science Institute, Yonsei University, Seoul 03722, Republic of Korea*

[⊥]*Chemistry Institute for Functional Materials, Pusan National University, Busan 46241, Republic of Korea*

* Correspondence to: hughkim@korea.ac.kr, jmchoi@pusan.ac.kr

Page

Supplementary Note. *in silico* prediction of each fragment's binding to the pre-fibrillary form of amyloid-β (1-42)··········4

Table S1. List of amyloid-β (1-42) fragments

Figure S1. AlphaFold prediction of the binding of each fragment to the prefibrillar form of amyloid-β (1-42)

Figure S2. Self-assembly of amyloid-β (1-42) fragments

Supplementary Note. Monitoring the effects of amyloid-β (1-42) fragments and designed peptide inhibitors on amyloid-β (1-42) aggregation ··········8

Figure S3. ThT fluorescence assay to monitor the self-assembly properties of P_a and P_b

Figure S4. Effects of amyloid-β (1-42) fragments (F_a and F_b) on the amyloid aggregation of amyloid-β (1-42)

Figure S5. Length distribution of amyloid-β (1-42) fibrils affected by the peptide inhibitor candidates based on TEM image analysis

Figure S6. Effects of designed peptides on fibrillar amyloid aggregation of 25 μM amyloid-β (1-42)

Supplementary Note. Investigating three key domains of amyloid-β (1-42)··········15

Figure S7. Three regions of interest in amyloid-β (1-42)

Figure S8. Three groups of theoretical ensembles based on the radius of gyration

Supplementary Note. Multidisciplinary biophysical analysis of amyloid-β (1-42)-inhibitor complexes ··········19

Figure S9. MS peaks after HDX of amyloid-β (1-42)

Figure S10. CCS calibration curve using standard proteins with known CCS values

Figure S11. AlphaFold predicted complex structures of amyloid-β (1-42) monomer

Figure S12. Root-mean-square deviation (RMSD) of amyloid-β (1-42)-inhibitor complex REMD simulation with respect to final structure

Figure S13. Contact probability of designed peptides with each hydrophobic domain

Figure S14. Concomitant bindings of P_{ab} to amyloid-β (1-42)

Figure S15. Representative molecular dynamics (MD) conformations of the amyloid-β (1-42)

Table S2. Binding free energy calculation results

Figure S16. ETD fragmentation pattern and ETD mass spectra

Supplementary Note. Examine the role of three key domains in amyloid-β (1-42) self-assembly using its point mutant I41N/A42N··32

Figure S17. ThT fluorescence assay for monitoring the amyloid aggregation kinetics of wild-type amyloid-β (1-42) and its mutant

Figure S18. Experimental collision cross section distributions of amyloid-β (1-42) mutant (I41N/A42N) and the complex ions with peptide inhibitor

Supplementary References··35

Supplementary Note. *In silico* prediction of binding of each fragment to the pre-fibrillary form of amyloid-β (1-42)

AlphaFold prediction of each fragment's binding to the pre-fibrillary form of amyloid-β (1-42). AlphaFold prediction of amyloid-β (1-42)-fragment and amyloid-β (1-42)-peptide complexes was performed using a locally installed version of the software downloaded from the GitHub repository. Source code is available at https://github.com/deepmind/alphafold. The AlphaFold parameters were multimer (model_preset) and full_dbs (db_preset).

Materials. All reagents were purchased from commercial suppliers and used as received, unless otherwise stated. Synthetic wild-type amyloid-β (1-42), its point mutant (I41N/A42N), its fragments, and peptide inhibitor candidates (purity > 95%) were purchased from Anygen (Gwangju, Republic of Korea). The concentration of amyloid-β (1-42) was determined using a UV-visible (UV-Vis) spectrophotometer (ε_{280} nm = 1490 $M^{-1} \cdot cm^{-1}$). Tris-HCl stock solution (1 M, pH 7.4) was purchased from Biosesang (Daejeon, Republic of Korea) and HPLC-grade water was purchased from JT Baker (Phillipsburg, NJ, USA). Thioflavin-T (ThT) was purchased from Sigma-Aldrich (St. Louis, MO, USA).

ThT fluorescence assay to investigate amyloid aggregation kinetics of F_a and F_b. ThT fluorescence assay was performed to observe the amyloid aggregation kinetics of the two fragments of amyloid-β (1-42) (Figure S2). Samples were incubated in 20 mM Tris-HCl buffer (pH 7.4) at 37 °C without agitation on a Synergy H1 microplate reader (BioTek, Winooski, VT, USA). The fluorescence intensity was measured using a microplate reader, and the error bars represent the standard deviation from three independent experiments.

Name	Notation	Amino acid sequence	Corresponding region
Fragment a	F_a	KLVFFAE	16-22
Fragment b	F_b	GSNKGAIIGLM	25-35
Fragment c	F_c	EFRHDSGYEVHHQK	3-16
Fragment d	F_d	VHHQKLVFFAEDVGSNK	12-28
Fragment e	F_e	AEDVGSNKGA	21-30
Fragment f	F_f	GAIIGLMVGGVV	29-40
Fragment g	F_g	IIGLM	31-35
Fragment h	F_h	DAEFRHDSGYE	1-11
Fragment i	F_i	DAEFRHDSGYEVHHQ	1-15
Fragment j	F_j	DAEFRHDSGYEVHHQK	1-16
Fragment k	F_k	DAEFRHDSGYEVHHQKLVFF	1-20
Fragment l	F_l	DAEFRHDSGYEVHHQKLVFFAED	1-23
Fragment m	F_m	DAEFRHDSGYEVHHQKLVFFAEDV	1-24
Fragment n	F_n	DAEFRHDSGYEVHHQKLVFFAEDVGSNK	1-28
Fragment o	F_o	DAEFRHDSGYEVHHQKLVFFAEDVGSNKGA	1-30
Fragment p	F_p	DAEFRHDSGYEVHHQKLVFFAEDVGSNKGAIIG	1-33
Fragment q	F_q	DAEFRHDSGYEVHHQKLVFFAEDVGSNKGAIIGL	1-34

Table S1. List of amyloid-β (1-42) fragments

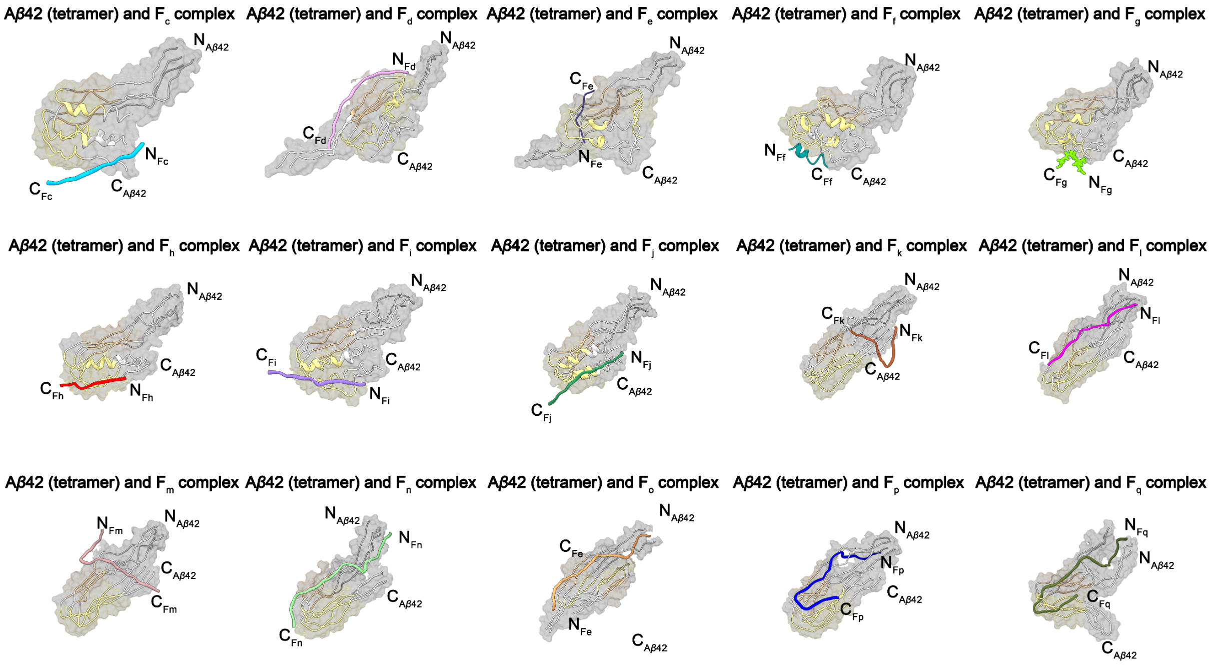

Figure S1. AlphaFold prediction of each fragment's binding to the prefibrillar form of amyloid-*β* (1-42). The central hydrophobic region of amyloid-*β* (1-42) (^{16}KLVFFAE^{22}) is colored tan, ^{25}GSNKGAIIGLM^{35} is colored khaki, and each fragment is plotted in different color. Amyloid-*β* (1-42) and each fragment's N- and C-termini are labeled.

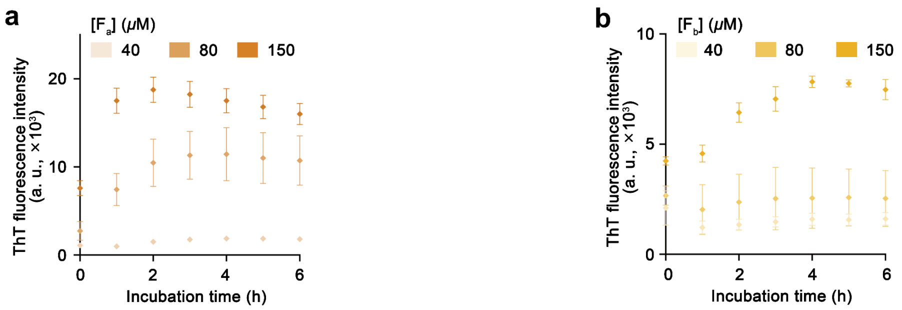

Figure S2. Self-assembly of amyloid-β (1-42) fragments (F_a and F_b) was monitored by thioflavin T fluorescence assay. The error bars represent the standard deviation from three independent experiments.

Supplementary Note. Effects of amyloid-β (1-42) fragments and designed peptide inhibitors on amyloid-β (1-42) aggregation.

Suppressed self-assembly properties of amyloid-β (1-42) fragments (F_a and F_b) by point mutation. We monitored the self-assembly properties of the peptide inhibitor candidates (P_a and P_b) (Figure S3). The results revealed that the point mutation suppressed the self-assembly of the two selected fragments of amyloid-β (1-42) (F_a and F_b).

Effects of amyloid-β (1-42) fragments (F_a and F_b) on the amyloid aggregation of amyloid-β (1-42). The F_a having no F19N point mutation did not show a decrease in fluorescence intensity but induced the delay in kinetics of amyloid-β (1-42) aggregation in a concentration dependent manner ($t_{1/2,\, A\beta42}$ = 20.46 ± 0.65 h, $t_{1/2,\, A\beta42\ w/\ 2\ \mu M\ Fa}$ = 21.02 ± 0.29 h, $t_{1/2,\, A\beta42\ w/\ 20\ \mu M\ Fa}$ = 22.11 ± 1.61 h, and $t_{1/2,\, A\beta42\ w/\ 50\ \mu M\ Fa}$ = 23.27 ± 0.47 h, respectively) (Figure S4). The kinetical delay of Aβ42 amyloid aggregation induced by P_a was more effective than F_a ($t_{1/2,\, A\beta42}$ = 20.93 ± 1.10 h, $t_{1/2,\, A\beta42\ w/\ 2\ \mu M\ Pa}$ = 22.61 ± 1.88 h, $t_{1/2,\, A\beta42\ w/\ 20\ \mu M\ Pa}$ = 23.59 ± 1.20 h, and $t_{1/2,\, A\beta42\ w/\ 50\ \mu M\ Pa}$ = 25.76 ± 0.95 h, respectively) (Figure 2a). The mixture of amyloid-β (1-42) and peptide F_b, which does not include the I32N point mutation, showed double sigmoidal growth in ThT fluorescence intensity (Figure S4).

Transmission electron microscopy (TEM) image analysis. To prepare samples for TEM analysis, we used a negative staining protocol with uranyl acetate purchased from Sigma-Aldrich. Uranyl acetate stock solution of 0.5 % w/v concentration was prepared in HPLC grade water and filtered with a 0.22 μm disposable syringe filter. The total concentration of Aβ peptides was 10 μM and the peptides were incubated for 24 h before being transferred to a 400-mesh formvar/carbon Cu(II) grid (Electron Microscopy Science, Hatfield, PA, USA). The incubated fibril samples (5 μL) were spotted onto a Cu(II) grid for 3 min at 20 °C and removed. The grids were washed twice immediately after removing the samples using 0.5 % *w*/*v* uranyl

acetate solution. Each sample was then stained with 5 μL uranyl acetate solution for one minute. The samples were dried for 4 h at 20 °C following removal of the staining solution.

Fibril length distribution. TEM images were further analyzed using an Able Image Analyzer (version 4.0) to determine the lengths of Aβ42 fibrils. Fibril length was defined as the end-to-end distance between the identifiable and negatively stained fibrils. The lengths of 200 different fibril species were measured manually. We measured the lengths of 200 fibrils from the TEM images, and the results showed that the peptide inhibitor candidate P_{ab} induced the formation of longer and more dispersed fibrils, indicating interference in the early aggregation stage (Figure S5).[1]

Effects of designed peptides (P_{ab}, P_a, and P_b) on the amyloid aggregation of amyloid-*β* (1-42) above the critical micelle concentration. In the early aggregation stages, all three designed peptides kinetically delayed fibrillar amyloid aggregation of amyloid-β (1-42), but only P_{ab} and P_a significantly suppressed the formation of amyloid aggregates at the endpoint (Figures 1 and 2). Since there have been reports of micelles-like oligomer formation above the critical concentration of amyloid-β (1-42) proteins, we investigated the suppression effect of designed peptides on amyloid aggregation above the critical micelle concentration (Figure S6).[2-3] The aggregation half time of the 25 μM amyloid-β (1-42) sample was 8.12 ± 0.04 h, while the inhibitor-treated samples took 20.18 ± 0.62 h, 18.00 ± 0.19 h, and 17.22 ± 0.91 h, respectively (for P_{ab}, P_a, and P_b) (Figure S6a). The thioflavin T fluorescence intensity was decreased at the endpoint when Pab or Pa was treated to 25 μM amyloid-β (1-42). Our findings show that even though the amyloid-β (1-42) protein concentration is increased, the suppressive effect of designed peptides persisted. We also performed transmission electron microscopy image analysis on the fibrils of amyloid-β (1-42) above CMC to visualize the thioflavin T-positive species in the thioflavin T incubation assay (Figure S6b). We confirmed that the

fibrillar amyloid aggregates were formed in our laboratory conditions, and the results agreed with the thioflavin T incubation assay result.

Cell cultures. SH-SY-5Y neuroblastoma cells were obtained from the Korean Cell Line Bank (Seoul, Republic of Korea) and cultured in a 1:1 mixture of Dulbecco's Modified Eagle Medium/F-12 Nutrient Mixture Ham (DMEM/F-12; WelGENE, Daegu, Republic of Korea) supplemented with 10% fetal bovine serum (FBS; Capricorn Scientific GmbH, Ebsdorfergrund, Germany) and 1% antibiotics (10,000 U/mL penicillin G, 10,000 μg/mL streptomycin, and 25 μg/mL amphotericin B; Hyclone, Logan, UT, USA). The antibiotics were sterilized prior to use by passage through a 0.2 μm filter. The cells were maintained at 37 °C in a humidified atmosphere containing 5 % CO_2.

Cell viability test among cells treated with Aβ42 fibrils and different peptide inhibitor candidates. We performed a modified thiazolyl blue tetrazolium bromide (MTT) assay to monitor cell viability in SH-SY-5Y neuroblastoma cells, a widely used cellular model for screening the cytotoxicity of amyloid proteins. Cell viability was assessed using the MTT assay. Aβ42 fibrils (final Aβ42 concentration 2 μM) were prepared during a 48-hour incubation at 37 °C and peptide inhibitor candidates were added at multiple concentrations (2 μM, 20 μM, and 50 μM). Cells (15,000 cells) were seeded in each well of a 96-well plate and incubated for 24 h. The SH-SY-5Y cells were treated with preformed Aβ42 fibrils for two days. The final Aβ42 was 0.2 μM. MTT solution was added to the medium and incubated for 3 h at 37 °C to form blue MTT-formazan products, which were assessed by measuring absorbance at 540 nm. Each set was tested in triplicate and the assay was repeated three times to confirm reproducibility.

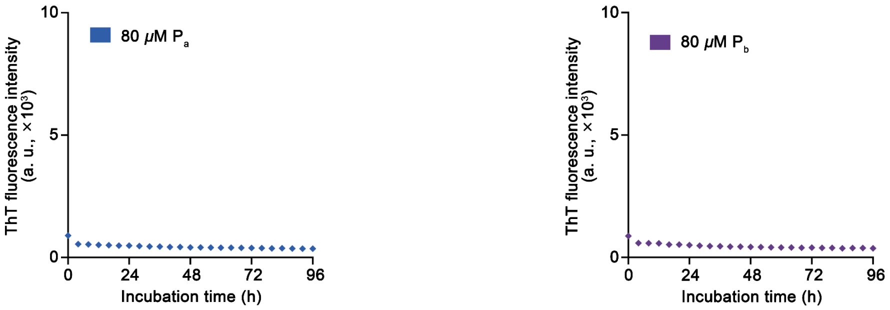

Figure S3. ThT fluorescence assay to monitor the self-assembly properties of P_a and P_b. Both the two fragments did not show an increment in ThT fluorescence intensity. The error bars represent the standard deviation from three independent experiments.

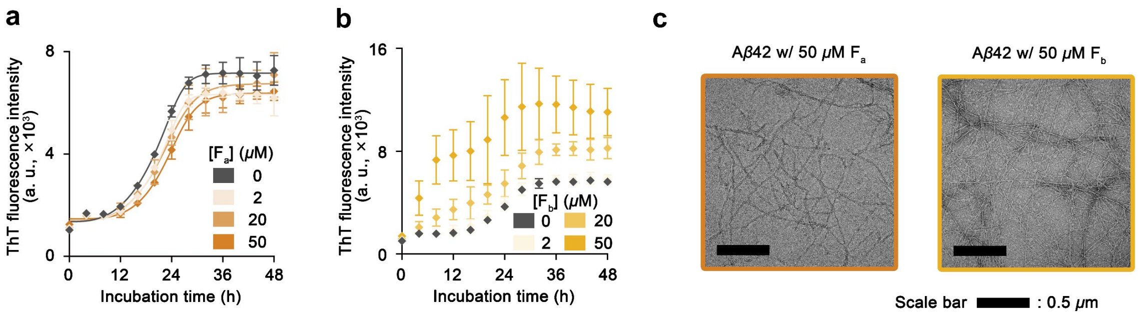

Figure S4. Effects of amyloid-β (1-42) fragments (F_a and F_b) on the amyloid aggregation of amyloid-β (1-42). The error bars represent the standard deviation from three independent experiments.

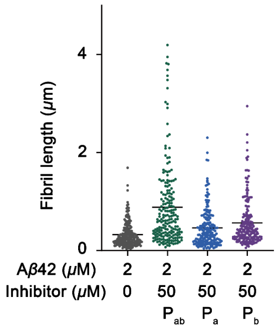

Figure S5. Length distribution of amyloid-*β* (1-42) fibrils affected by the peptide inhibitor candidates based on TEM image analysis. The designed peptides induced the formation of longer and more dispersed fibrils.

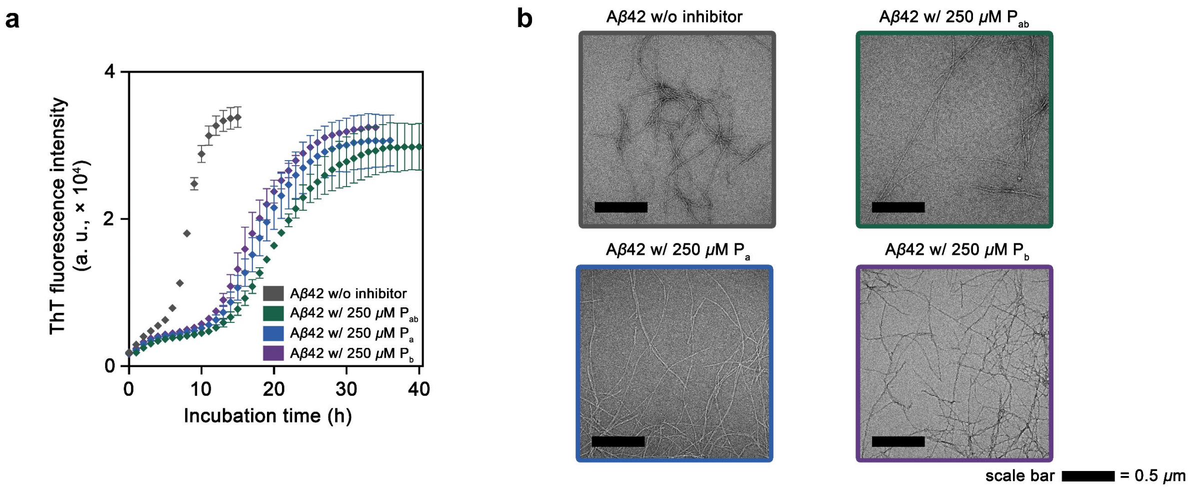

Figure S6. Effects of designed peptides on fibrillar amyloid aggregation of 25 μM amyloid-β (1-42). (a) Thioflavin T fluorescence assays were used to trace the aggregation kinetics of the mixture of 25 μM amyloid-β (1-42) and three designed peptides (250 μM). (b) Transmission electron microscopy images of amyloid-β (1-42) fibrils in mixtures of amyloid-β (1-42) and designed peptides. The error bars represent the standard deviation from two independent experiments.

Supplementary Note. Investigation of three key domains of amyloid-β (1-42)

Protein solubility calculation using the CamSol method. To approximate the protein solubility of amyloid-β (1-42) (Aβ42), we calculated its solubility at neutral pH (pH 7) using the CamSol web server (http://www-vendruscolo.ch.cam.ac.uk/camsolmethod.html). We employed 'CamSol Intrinsic', without a template protein structure.

SAXS experiments. SAXS measurements were performed to obtain the structural details at the monomer level. SAXS experiments were performed using the 4C SAXS II beamline of the Pohang Accelerator Laboratory (PAL). The concentration of Aβ42 was adjusted to 2 mg/mL in 20 mM Tris-HCl buffer (pH 7.4) and the temperature was maintained at 20 °C during exposure to the X-ray beam. The sample-to-detector distance was set to 1 m. During each of the three independent experiments, the scattering patterns were recorded for 3 s and measured ten times. The Guinier approximation of the SAXS profiles was conducted to obtain the radius of gyration (R_g) (Supplementary Equation 1), where q is the scattering vector and $I(q)$ is the scattering intensity at q.

$$\ln[I(q)] = \ln[I(0)] - R_g^2/3q^2 \quad - \quad (1)$$

Kratky analysis of the SAXS profiles ($I(q)\cdot q^2$ as a function of q showed that the conformation of the Aβ42 protein was disordered. The Kratky plot is made dimensionless by using the product of the scattering vector q and R_g instead of q ($I(q)/I(0)\cdot(q\cdot R_g)^2$ as a function of $q\cdot R_g$).

External pool generation of wild-type Aβ42 for EOM analysis We performed an ensemble optimization method (EOM) analysis using the external pool (10,000 structures) acquired from replica-exchange MD (REMD) simulation with the CHARMM36m force field and the General Born implicit solvation model.[4-5] The initial structure of the wild-type Aβ42 monomer was obtained from the cryo-EM structure of Aβ42 fibrils (PDB ID:5OQV) from the Protein Data Bank (www.rcsb.org).[6] Five replicas (T = 400, 424, 450, 476, and 500 K) were

used to obtain an average exchange probability of ~0.2. Each replica was simulated for 20 ns, and 10,000 MD-simulated random conformations were extracted from replicas for EOM analysis. Simulation temperatures were generated by the 'remd-temperature-generator' web server (http://virtualchemistry.org/remd-temperature-generator/).[7] Fifty structures were selected from the EOM analysis to fit the small-angle X-ray scattering (SAXS) profile.

Representative structures of the A*β*42 protein. Representative conformations were obtained by EOM analysis with 10,000 MD-simulated conformations. The size of the ensemble was fixed at 50 curves per ensemble, and repetitions were disallowed. The optimized result fitted well with the experimental R_g value ($R_{g,\ theo}$ = 22.40 ± 3.19 Å and $R_{g,\ exp}$ = 22.81 ± 1.25 Å, respectively) (Figure 3b).

Three groups of theoretical ensembles of the A*β*42 protein. We divided the theoretical ensembles of A*β*42 into three groups based on the radius of gyration (Figure S8). Compact conformations among the 50 EOM structures (lowest one-third radius of gyration) are referred to as group 1, intermediates as group 2, and extended conformers (largest one-third radius of gyration) as group 3. The solvent-accessible surface area was analyzed using the DSSP program.[8]

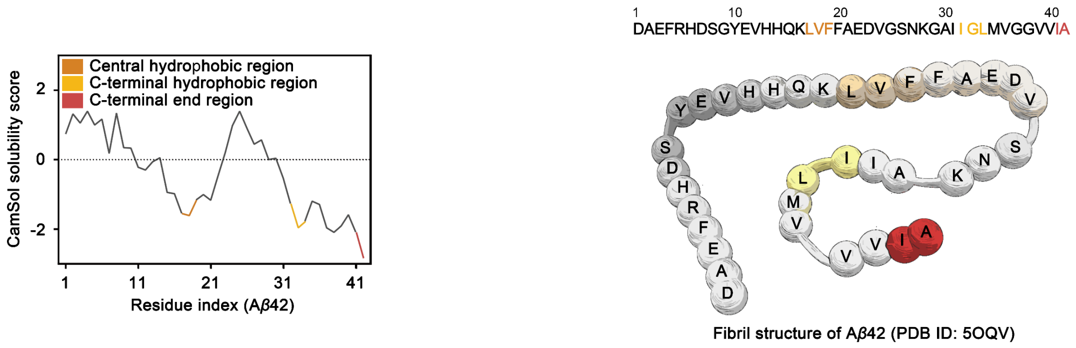

Figure S7. Three regions of interest in amyloid-β (1-42) (central hydrophobic region, ^{17}LVF^{19}, Φ_1; C-terminal hydrophobic region, ^{32}IGL^{34}, Φ_2; and C-terminal end, ^{41}IA^{42}, Φ_3).

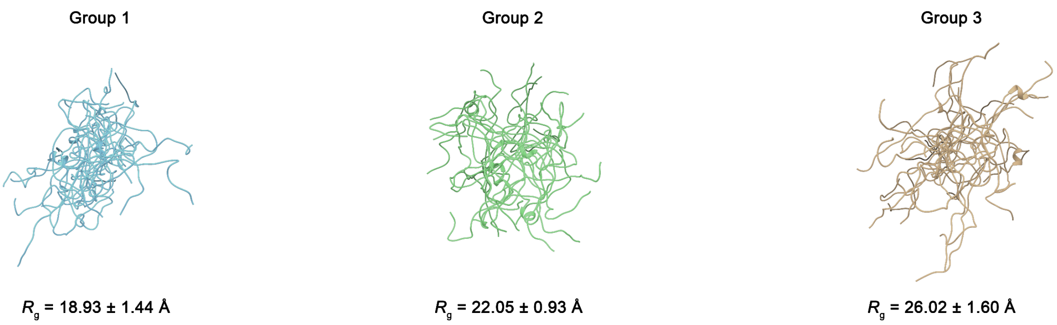

Figure S8. Three groups of theoretical ensembles based on the radius of gyration. Compact conformations among the 50 EOM structures (lowest one-third radius of gyration) are referred to as group 1, intermediates as group 2, and extended conformers (largest one-third radius of gyration) as group 3.

Supplementary Note. Multidisciplinary biophysical analysis of amyloid-β (1-42)-inhibitor complexes

Hydrogen deuterium exchange combined with MS. Freshly prepared Aβ42 and the three peptide inhibitor candidates were mixed and diluted with deuteration solvent. Samples were exposed to D_2O (99.9% atom D) for 1 s and directly quenched with formic acid (0.1% of total volume). After addition of the quenching agent, the samples were directly injected into a Synapt G2-Si mass spectrometer (Waters, Manchester, UK). The volume ratios for the deuteration solvent and non-deuteration solvent was 3 to 2, and the final concentrations of Aβ42 and peptide inhibitor candidates were 10 μM and 50 μM, respectively. Backward corrections were performed using the fully deuterated samples. The volume ratio for the deuteration solvent and non-deuteration solvent was adjusted to be the same as that for normal HDX, except for the addition of the deuteration solvent at the beginning. The fully deuterated samples underwent backward exchange with the non-deuterated solvent for the same amount of time as the normal HDX. All measurements were performed in triplicate, and the deuteration level was determined using the equation below (Supplementary equation 2).

$$(Deuteration\ level) = \frac{(the\ number\ of\ exxchanged\ deuteriums)+(the\ number\ of\ backward\ exchanged\ hydrogens)}{(total\ number\ of\ exchangable\ hydrogens\ in\ proteins)} \times 100(\%)$$

(2)

ESI-MS parameters. A Synapt G2-Si HDMS quadrupole time-of-flight (Q-TOF) mass spectrometer (Waters, UK, Manchester) was used for the MS analysis. In the positive ion mode, source temperature of 80 °C, a capillary voltage of 2.5 kV, a desolvation temperature of 150 °C, and a cone voltage of 20 V were set as the parameters for ESI source.

Gas-phase structural analysis using IM-MS. To conduct additional structural analysis, the structural dynamics of natural and mutant ions in the gas phase were investigated using IM-

MS, which can analyze gas-phase structures by determining gaseous ion collision cross-sections (CCSs). In the IM spectra, we observed an extended conformation from the Aβ42-P$_a$ and Aβ42-P$_b$ ions (+4 charge state), which was not present in the Aβ42-P$_{ab}$ ion (Figure 4b). We performed MD simulations of the peptides *in vacuo* using representative structures obtained from the REMD trajectories as the initial structures. Gas-phase structural analyses were then performed based on theoretical CCS calculations of the simulated structures that matched the experimental CCS values. The simulated structures indicated that desolvation of the extended structures of Aβ42-P$_a$ and Aβ42-P$_b$ ions corresponded to the extended conformer in the gas phase (Figure 4c). In contrast, structural collapse during desolvation induced a compact conformation of the complex ions.

IM-MS analysis. IM-MS experiments were performed using a Waters Synapt G2-Si HDMS quadrupole time-of-flight (Q-TOF) mass spectrometer (Waters, Manchester, UK) with travelling wave ion mobility spectrometry (TWIMS) capability. Each Aβ42 (10 μM) in HPLC-grade H_2O (0.1% formic acid) with or without peptide inhibitors (50 μM) was sprayed into the electrospray ionization (ESI) source at a flow rate of 10 μL/min. The capillary voltage was 2.5 kV, and the source temperature was 80 °C. The gas flow rates for the helium and drift cells were 180 and 90 mL/min, respectively, providing a pressure of 3.19 mbar in the drift cell. The CCSs of the Aβ42 conformers were calibrated following the procedure developed by Ruotolo *et al.* and standards reported by Bush *et al.*[9]

Equilibrium ensembles of Aβ42-inhibitor complexes. Based on the five 4.95-μs REMD simulations (150 ns per replica) with the CHARMM36m force field for each initial structure (Figure S11), we calculated the interchain contact probabilities of Aβ42 and each inhibitor using the GROMACS software package (version 2020.4).[10-11] The initial structures were obtained from AlphaFold predictions. We adopted the simulation protocol method from Man *et al.* (2017).[12] Simulations were performed at pH 7 in a cubic TIP3P water box containing 20

mM Na^+ and Cl^- ions.[13] The GROMACS software package (version 2020.4) with the SHAKE algorithm allowed a time step of 2 fs.[14] For electrostatic interactions, we used the particle mesh Ewald (PME) method with a cutoff of 1.2 nm, and for the van der Waals interactions, the cutoff was set to 1.2 nm, and a velocity-rescaling thermostat was employed.[15-16] The REMD was carried out with 33 replicas from 300 K to 400 K, and exchanges between replicas were attempted every 2 ps with an exchange probability of 0.15. The RMSD of the final structure merged after 10 ns (Figure S12). The interchain distances between the β carbons of each side chain (α carbon for glycine) and the residues were *in contact* if the distance between the two designated atoms was smaller than 0.8 nm.

R_g distribution of Aβ42 and representative MD simulated structures. R_g distribution of Aβ42 is calculated using gmx gyrates module. Representative structures of Aβ42 were obtained from the simulated REMD structure (Aβ42-P_b). The structures in the figures were modelled using UCSF Chimera.[17-18]

Binding free energy calculation. We employed umbrella sampling to calculate the binding free energy of peptide inhibitor candidates for Aβ42, as described by Lemkul *et al.* (2010).[19] The models for the Aβ42-inhibitor complexes chosen for this *in silico* study were the AlphaFold predicted structures. Simulations were conducted using the GROMACS software package (version 2020.4), and the CHARMM36m parameter set was applied to all species in the simulated system.[11] To generate equilibrated starting structures for the center-of-mass pulling simulations, each structure was placed in a TIP3P water box, in which 20 mM NaCl was added, including neutralizing counterions.[13] Protein and non-protein atoms were coupled to separate temperature-coupling baths, and the temperature was maintained at 310 K using the V-rescale method.[16] In which a 10 ns MD simulation was performed for a total simulation time of 300 ns utilized for umbrella sampling. Analysis of the results was performed using the weighted histogram analysis method.[20-21] The potential of the mean force between the two

chains was calculated to investigate the binding free energy of peptide inhibitor candidates to Aβ42. When the peptide inhibitor candidate was pulled away from Aβ42, the binding free energy increased, indicating the presence of attractive intermolecular interactions between the peptide inhibitor candidate and Aβ42.

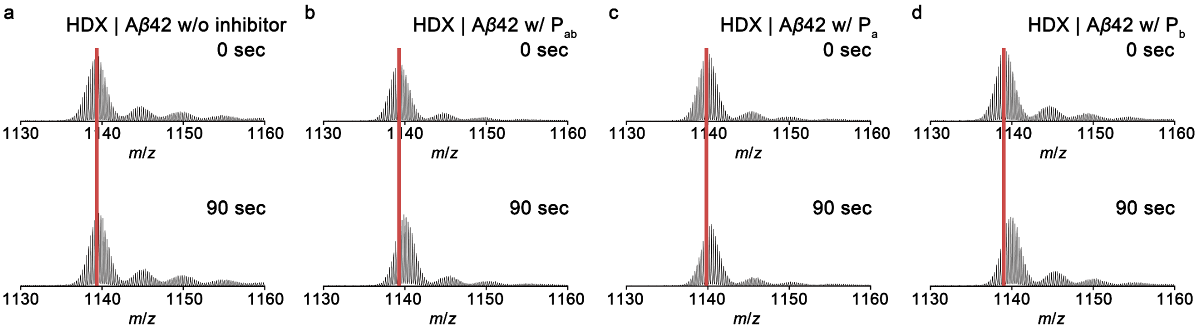

Figure S9. Mass spectrum (MS) peaks after hydrogen-deuterium exchange (HDX) of amyloid-β (1-42) (4^+) (a) without peptide inhibitor, (b) with P_{ab}, (c) with P_a, and (d) with P_b.

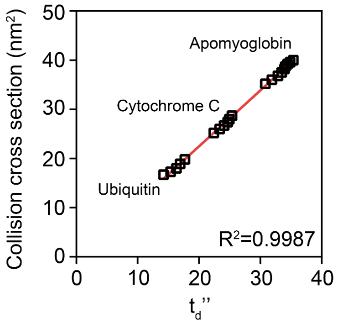

Figure S10. Collision cross-section (CCS) calibration curve using standard proteins with known CCS values.

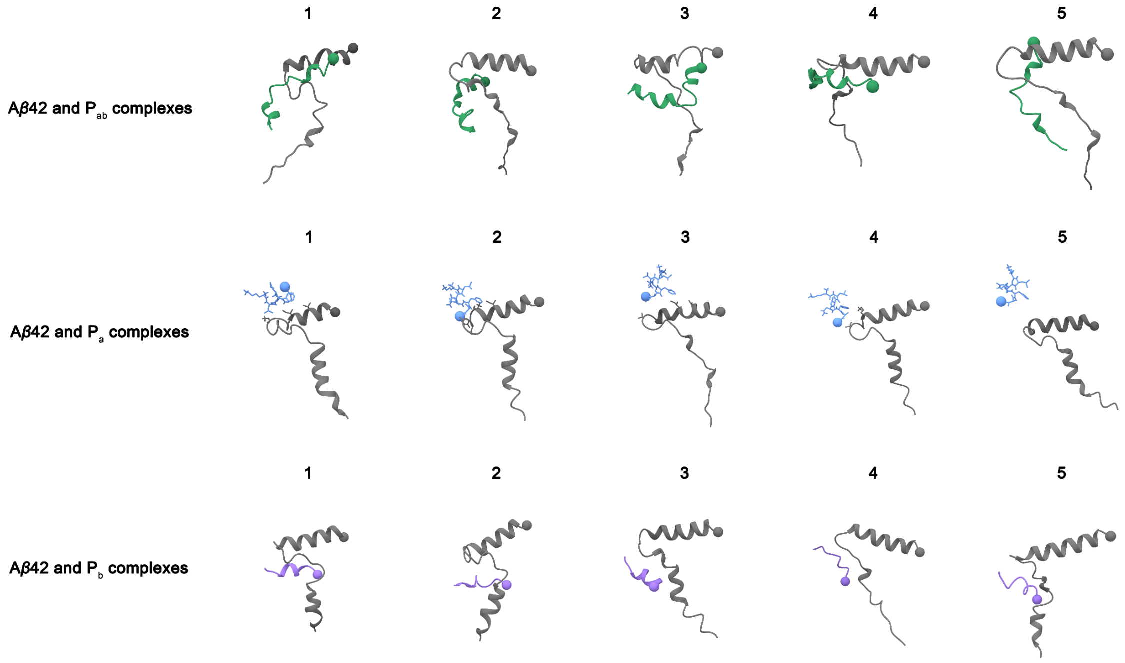

Figure S11. AlphaFold-predicted complex structures of amyloid-*β* (1-42) (A*β*42) monomer.

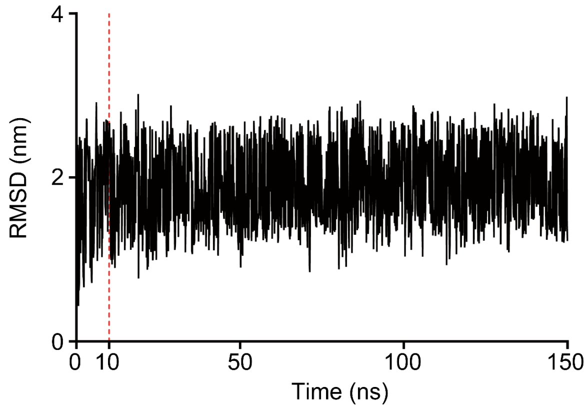

Figure S12. Root-mean-square deviation (RMSD) of amyloid-β (1-42)-inhibitor complex REMD simulation with respect to final structure. The RMSD of the final structure merged after 10 ns.

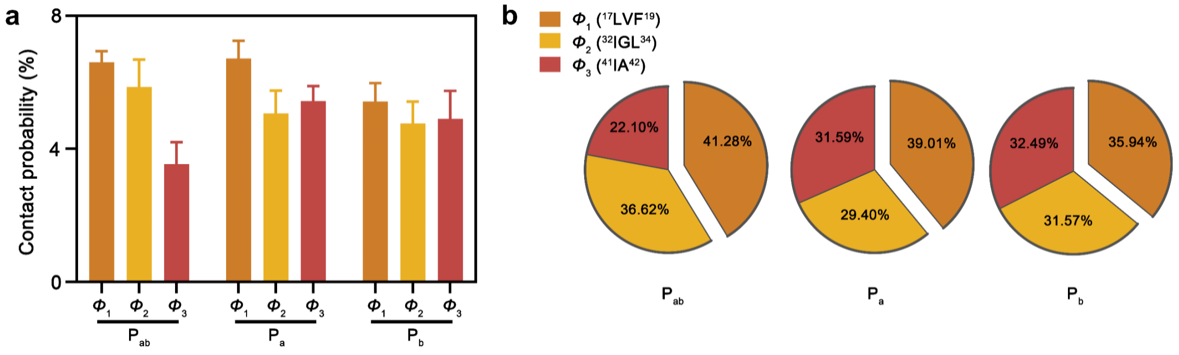

Figure 13. Contact probability of designed peptides with each hydrophobic domain. (a) Contact probabilities derived from molecular dynamics simulation trajectories, and (b) relative abundances of contacts to the three hydrophobic domains. The error bars represent the standard deviation.

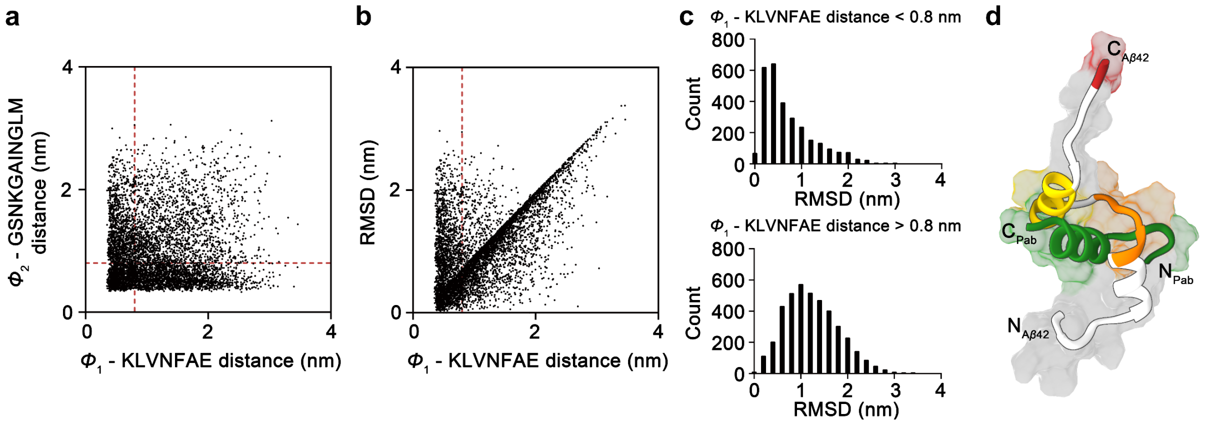

Figure S14. Concomitant bindings of P_{ab} to amyloid-β (1-42). (a) The distance between Φ_2 domain of amyloid-β (1-42) protein and GSNKGAINGLM of P_{ab} (corresponding to P_b) versus the distance between the Φ_1 domain of amyloid-β (1-42) protein and KLVNFAE of P_{ab} (corresponding to P_a). (b) Calculated root-mean-square deviation (RMSD) values for the two distances in each data point. (c) The population distribution of calculated RMSD values based on whether the distance between the Φ_1 domain of amyloid-β (1-42) protein and KLVNFAE of P_{ab} was smaller or larger than 0.8 nm. (d) Representative structure of amyloid-β (1-42) protein and P_{ab} complex obtained from the molecular dynamics simulation trajectory.

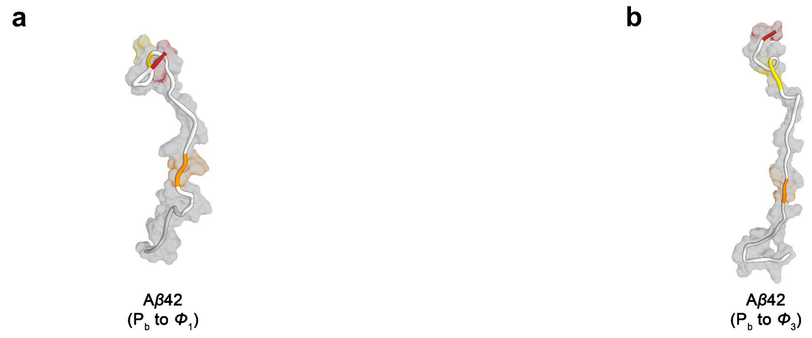

Figure S15. Representative molecular dynamics (MD) conformations of the A*β*42 obtained from A*β*42-P_b complexes with which the designed peptide interact with (a) Φ_1 and (b) Φ_3.

Name	Initial structure 1	Initial structure 2	Initial structure 3	Initial structure 4	Initial structure 5
P_{ab}	27.03 kJ/mol	19.95 kJ/mol	40.92 kJ/mol	15.68 kJ/mol	10.83 kJ/mol
P_a	13.04 kJ/mol	12.76 kJ/mol	8.91 kJ/mol	6.92 kJ/mol	3.28 kJ/mol
P_b	41.03 kJ/mol	15.48 kJ/mol	9.06 kJ/mol	11.49 kJ/mol	49.20 kJ/mol

Table S2. Binding free energy calculation results

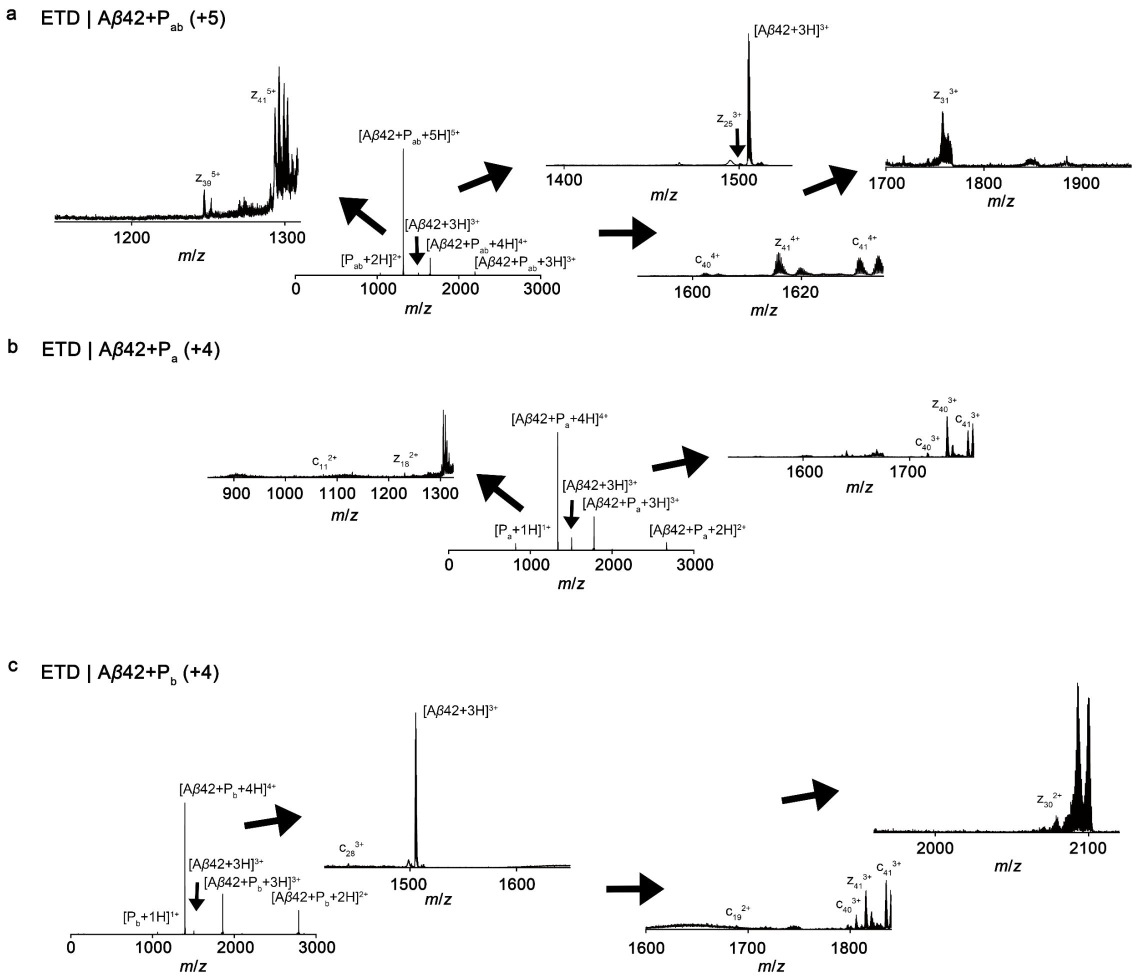

Figure S16. Electron transfer dissociation (ETD) fragmentation pattern and ETD mass spectra of (a) amyloid-β (1-42)-P_{ab}, (b) amyloid-β (1-42)-P_a, and (c) amyloid-β (1-42)-P_b complex.

Supplementary Note. Examining the role of three key domains in amyloid-*β* (1-42) self-assembly using its point mutant I41N/A42N

Comparison of amyloid aggregation in mixtures of amyloid-*β* (1-42) I41N/A42N point mutant and three designed inhibitor candidates. We used a point mutant of A*β*42 (I41N/A42N) to investigate the role of the three key domains in A*β*42 self-assembly. We performed the ThT assay, TEM image analysis, and IM-MS experiments as described above, except for the concentration of A*β*42 I41N/A42N (10 μM). The designed peptide inhibitor candidates were applied in 1:1, 1:5, or 1:25 molar ratios to monitor amyloid aggregation.

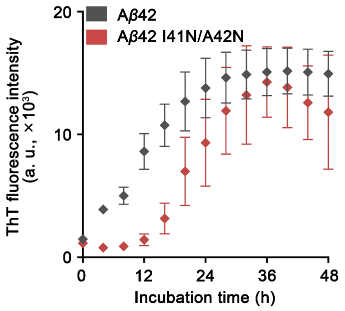

Figure S17. ThT fluorescence assay for monitoring the amyloid aggregation kinetics of wild-type amyloid-*β* (1-42) (A*β*42) and its mutant (I41N/A42N).

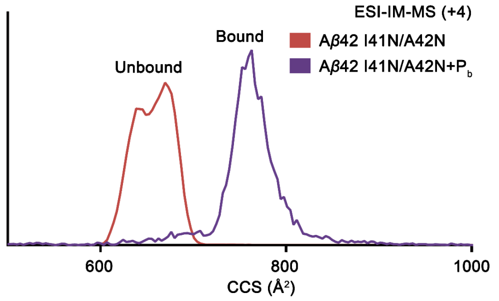

Figure S18. Experimental collision cross-section distributions of amyloid-β (1-42) mutant (I41N/A42N) and the complex ions with peptide inhibitor P_b.

1. Choi, T. S.; Lee, H. H.; Ko, Y. H.; Jeong, K. S.; Kim, K.; Kim, H. I., Nanoscale Control of Amyloid Self-Assembly Using Protein Phase Transfer by Host-Guest Chemistry. *Sci. Rep.* **2017**, *7*, 5710.
2. Yong, W.; Lomakin, A.; Kirkitadze, M. D.; Teplow, D. B.; Chen, S. -H.; Benedek, G. B., Structure determination of micelle-like intermediates in amyloid β-protein fibril assembly by using small angle neutron scattering. *Proc. Nat. Acad. Sci. U.S.A.* **2002**, *99* (1), 150-154.
3. Morel, B.; Carrasco, M. P.; Jurado, S.; Marco, C.; Conejero-Lara, F., Dynamic micellar oligomers of amyloid beta peptides play a crucial role in their aggregation mechanisms. *Phys. Chem. Chem. Phys.* **2018**, *20* (31), 20597-20614.
4. Schaefer, M.; Bartels, C.; Karplus, M. Solution conformations and thermodynamics of structured peptides: molecular dynamics simulation with an implicit solvation model. *J. Mol. Biol.* **1998,** *284*, 835–48.
5. Onufriev, A.; Bashford, D.; Case, D. A. Exploring protein native states and large-scale conformational changes with a modified generalized born model. *Proteins* **2004,** *55*, 383–94.
6. Gremer, L.; Schölzel, D.; Schenk, C.; Reinartz, E.; Labahn, J.; Ravelli, R. B. G.; Tusche, M.; Lopez-Iglesias, C.; Hoyer, W.; Heise, H.; Willbold, D.; Schröder, G. F. Fibril structure of amyloid-β(1–42) by cryo–electron microscopy. *Science* **2017,** *358*, 116–119.
7. Patriksson, A.; van der Spoel, D. A temperature predictor for parallel tempering simulations. *Phys. Chem. Chem. Phys.* **2008,** *10*, 2073–2077.
8. Kabsch, W.; Sander, C. Dictionary of protein secondary structure: pattern recognition of hydrogen-bonded and geometrical features. *Biopolymers* **1983,** *22*, 2577–637.
9. Bush, M. F.; Hall, Z.; Giles, K.; Hoyes, J.; Robinson, C. V.; Ruotolo, B. T. Collision Cross Sections of Proteins and Their Complexes: A Calibration Framework and Database for Gas-Phase Structural Biology. *Anal. Chem.* **2010,** *82*, 9557–9565.
10. Van Der Spoel, D.; Lindahl, E.; Hess, B.; Groenhof, G.; Mark, A. E.; Berendsen, H. J. GROMACS: fast, flexible, and free. *J. Comput. Chem.* **2005,** *26*, 1701–1718.
11. Huang, J.; Rauscher, S.; Nawrocki, G.; Ran, T.; Feig, M.; de Groot, B. L.; Grubmüller, H.; MacKerell, A. D. CHARMM36m: an improved force field for folded and intrinsically disordered proteins. *Nat. Methods* **2017,** *14*, 71–73.
12. Man, V. H.; Nguyen, P. H.; Derreumaux, P. High-Resolution Structures of the Amyloid-β 1–42 Dimers from the Comparison of Four Atomistic Force Fields. *J. Phys. Chem. B* **2017,** *121*, 5977–5987.
13. Jorgensen, W. L.; Chandrasekhar, J.; Madura, J. D.; Impey, R. W.; Klein, M. L. Comparison of simple potential functions for simulating liquid water. *J. Chem. Phys.* **1983,** *79*, 926–935.
14. Ryckaert, J. P.; Ciccotti, G.; Berendsen, H. J. C. Numerical integration of the cartesian equations of motion of a system with constraints: molecular dynamics of n-alkanes. *J. Comput. Phys.* **1977,** *23*, 327–341.
15. Essmann, U.; Perera, L.; Berkowitz, M. L.; Darden, T.; Lee, H.; Pedersen, L. G. A smooth particle mesh Ewald method. *J. Chem. Phys.* **1995,** *103*, 8577–8593.
16. Bussi, G.; Donadio, D.; Parrinello, M. Canonical sampling through velocity rescaling. *J. Chem. Phys.* **2007,** *126*, 014101.
17. Pettersen, E. F.; Goddard, T. D.; Huang, C. C.; Couch, G. S.; Greenblatt, D. M.; Meng, E. C.; Ferrin, T. E. UCSF Chimera--a visualization system for exploratory research and analysis. *J. Comput. Chem.* **2004,** *25*, 1605–1612.

18. Sanner, M. F.; Olson, A. J.; Spehner, J. C. Reduced surface: an efficient way to compute molecular surfaces. *Biopolymers* **1996,** *38*, 305–320.
19. Lemkul, J. A.; Bevan, D. R. Assessing the Stability of Alzheimer's Amyloid Protofibrils Using Molecular Dynamics. *J. Phys. Chem. B* **2010,** *114*, 1652–1660.
20. Kumar, S.; Bouzida, D.; Swendsen, R. H.; Kollman, P. A.; Rosenberg, J. M. The weighted histogram analysis method for free-energy calculations on biomolecules. I: The method. *J. Comput. Chem.* **1992,** *13*, 1011–1021.
21. Hub, J. S.; de Groot, B. L.; van der Spoel, D. g_wham-A Free Weighted Histogram Analysis Implementation Including Robust Error and Autocorrelation Estimates. *J. Chem. Theory Comput.* **2010,** *6*, 3713–3720.
